# Development of Mathematical Model for Understanding Microcirculation in Diabetic Foot Ulcers Based on Ankle–Brachial Index

**DOI:** 10.3390/bioengineering12020206

**Published:** 2025-02-19

**Authors:** Ana Karoline Almeida da Silva, Gustavo Adolfo Marcelino de Almeida Nunes, Rafael Mendes Faria, Mário Fabrício Fleury Rosa, Lindemberg Barreto Mota da Costa, Newton de Faria, Adson Ferreira da Rocha, José Carlos Tatmatsu-Rocha, Suelia de Siqueira Rodrigues Fleury Rosa

**Affiliations:** 1 Postgraduate Programme in Mechatronic Systems, Mechanical Engineering Department, Campus Darcy Ribeiro, University of Brasilia, Brasilia 70910-900, DF, Brazil; gusnunes@gmail.com (G.A.M.d.A.N.); sdf73@cornell.edu (S.d.S.R.F.R.); 2 Department of Electrical Engineering, Federal Institute of Education, Science and Technology of Triângulo Mineiro, Campus Paracatu, Paracatu 38603-402, MG, Brazil; rafaelmendes@iftm.edu.br; 3 Postgraduate Programme in Biomedical Engineering, Faculty of Science and Engineering Technologies (FCTE), University of Brasilia, Gama 72444-240, DF, Brazil; mariorosafleury@gmail.com (M.F.F.R.); bergbmota1515@gmail.com (L.B.M.d.C.); adson@unb.br (A.F.d.R.); 4 Meinig School of Biomedical Engineering, Master of engineering (M.Eng.) Program, Cornell University, Ithaca, NY 14853, USA; newton.defaria@cornell.edu; 5 Graduate Program on Electrical Engineering, Department of Electrical Engineering, University of Brasilia, Darcy Ribeiro 70910-900, DF, Brazil; 6 Postgraduate Program in Physiotherapy in Functionality, Federal University of Ceará, Fortaleza 60430-275, CE, Brazil; tatmatsu@ufc.br

**Keywords:** angiogenesis, biomedical, computational biology, computer simulation, diabetic foot, microvessels, bond graph, particle swarm optimization (PSO)

## Abstract

This study proposes an innovative mathematical model for assessing microcirculation in patients with diabetic ulcers, using the ankle–brachial index (ABI). The methodology combines Bond Graph (BG) modeling and Particle Swarm Optimization (PSO), enabling a detailed analysis of hemodynamic patterns in a pilot sample of three patients. The results revealed a correlation between ulcer size and reduced ABI values, suggesting that deficits in microcirculation directly impact the severity of lesions. Furthermore, despite variations in ABI values and arterial pressures, all patients exhibited high capillary resistance, indicating difficulties in microcirculatory blood flow. The PSO-optimized parameters for the capillary equivalent circuit were found to be $R_{1}=89.784\;\Omega$, $R_{2}=426.55\;\Omega$, L=27.506H, and C=0.00040675F, which confirms the presence of high vascular resistance and reduced compliance in the microvascular system of patients with diabetic foot ulcers. This quantitative analysis, made possible through mathematical modeling, is crucial for detecting subtle changes in microcirculatory dynamics, which may not be easily identified through conventional pressure measurements alone. The increased capillary resistance observed may serve as a key indicator of vascular impairment, potentially guiding early intervention strategies and optimizing diabetic ulcer treatment. We acknowledge that the sample size of three patients represents a limitation of the study, but this number was intentionally chosen to allow for a detailed and controlled analysis of the variables involved. Although the findings are promising, additional experimental validations are necessary to confirm the clinical applicability of the model in a larger patient sample, thus solidifying its relevance in clinical practice.

## 1. Introduction

Diabetes mellitus (DM) is a major global health concern that leads to severe vascular complications, including diabetic foot ulcers (DFUs) [1,2,3]. According to the International Working Group on the Diabetic Foot (IWGDF), diabetic foot ulcers (DFUs) arise from multiple factors, including peripheral neuropathy (PN), peripheral arterial disease (PAD), infections, osteoarthropathy, and gangrene. These interconnected factors contribute to the onset and worsening of the condition. Vascular issues related to DFUs can impact large vessels (arteries and veins), small vessels (arterioles and venules), and microcirculation. A key aspect of this condition is the alteration in blood flow, which significantly affects the tissue repair process [4,5,6].

The study of microcirculation, especially in individuals with diabetes, is crucial for the early detection of vascular complications and for the proper management of the disease. Endothelial dysfunction, which represents an early stage in the development of atherosclerosis, is often present in diabetic patients and is an important factor in increasing cardiovascular risk [7,8]. Additionally, microvascular dysfunction is strongly associated with impaired wound healing. Poor tissue perfusion hampers the delivery of essential nutrients and oxygen for the healing process. In more severe cases, the deterioration of microcirculation can also result in dysfunction in vital organs, such as in the case of cardiac and vascular complications, which increases the risk of cardiovascular events like heart attacks and strokes. Therefore, the evaluation and monitoring of microcirculation in diabetic patients are essential for predicting, preventing, and effectively treating these complications [9].

The ankle–brachial index (ABI) is a widely used non-invasive tool for diagnosing PAD [10]. However, while the ABI effectively detects macrovascular obstructions, it does not directly assess the microvascular alterations that influence wound healing. This limitation underscores the need for alternative methods for evaluating capillary-level perfusion and its role in DFU progression [4].

Clinical tests, such as the ABI, while useful, do not provide a complete picture of the processes occurring at the microvascular level. For instance, while the ABI and transcutaneous partial pressure of oxygen (TcPO2) can indicate the presence of arterial compromise, they do not directly capture the biological and molecular factors that influence angiogenesis, microvascular perfusion, and tissue regeneration. This gap in information can hinder the analysis and interpretation of clinical results, limiting the effectiveness in managing diabetic ulcers and in the planning of therapeutic interventions [11,12].

Advanced techniques for evaluating microcirculation, including laser Doppler or near-infrared spectroscopy, provide detailed information on tissue perfusion, but they are expensive due to the sophisticated equipment required and the need for specialized training of professionals to use them correctly. Additionally, these methods require cutting-edge technology and constant maintenance, which can significantly increase costs, making them inaccessible in many regions or healthcare settings, especially in countries with limited resources [7,8,13].

The collection of hemodynamic data in capillaries generally requires invasive methods such as biopsies or catheterization, which are often not feasible or ethical. This limitation compromises the ability of clinicians and researchers to obtain detailed and accurate information directly from patients. Furthermore, diabetic patients with wounds can quickly develop complications, and invasive approaches may increase the risks of morbidity and mortality [14,15]. As a result, the lack of access to these technologies may pose a barrier to the proper management of diabetes-related complications, such as foot ulcers, thus highlighting the need to explore more accessible alternatives. Simpler, non-invasive methods, such as the ABI and TcPO2 measurements, remain essential, but their ability to provide comprehensive information on the microcirculatory state is still limited; this may require new technological solutions that are both effective and financially feasible for broader adoption [7,8,13].

Recent advances in computational modeling have enabled the simulation of microvascular hemodynamics, addressing gaps left by traditional diagnostic tools. Bond Graph(BG) modeling, a technique based on biophysical principles, allows for the representation of capillary blood flow dynamics through equivalent electrical circuits [16,17,18,19]. This approach provides a quantitative framework for understanding microcirculatory impairments in DFUs.

This study proposes a Bond Graph-based mathematical model for analyzing microcirculatory dynamics in DFUs, which correlates ABI values with capillary-level perfusion patterns. Using data from the three patients with distinct ABI classifications, we explore how the ABI reflects microvascular dysfunction. While the small sample size limits broad conclusions, this study serves as a proof-of-concept, laying the groundwork for future validation with larger datasets.

In silico data and mathematical modeling have significantly advanced diabetic wound research, overcoming challenges in direct patient analysis. The global push to reduce animal testing has led to alternative methods, though animal models remain crucial in areas like therapeutic development. Given the complexity of biological systems, safety and efficacy assessments cannot rely solely on animal testing. Instead, approaches such as in silico modeling, in vitro methods, omics technologies, and organ-on-a-chip technology offer viable alternatives. Integrated Approaches for Testing and Assessment (IATAs) combine these methods, enhancing our understanding of chemical interactions while minimizing animal experimentation [20,21,22].

This investigation is based on a limited sample of data obtained from a randomized clinical trial. To enhance the model’s accuracy, the BG approach is combined with Particle Swarm Optimization (PSO) algorithm, which has proven effective in optimizing parameters in complex models, includes applications in hemodynamics. PSO has been widely used in various biomedical contexts, such as in glucose prediction [23], as well as in optimization algorithms assisted by surrogate models, which combine global and local models to improve the accuracy and efficiency of optimal solution searches [24]. These advancements reinforce the applicability of PSO in modeling complex physiological phenomena, ensuring its relevance for the analysis and prediction of hemodynamic variables.

Based on the article [25], it is possible to reinforce the idea that small samples in the mathematical modeling of microcirculation contain essential information for validating hypotheses before applying them to larger cohorts. The article emphasizes that the mathematical modeling of microcirculation allows for a better understanding of the interconnected processes in metabolism and the identification of causes of microcirculatory disturbances. Additionally, it mentions that many initial models use simplified representations, such as a single representative capillary, allowing for the capture of important effects without the need for complex simulations of the entire capillary network [25].

We acknowledge that the sample of three patients represents a limitation of the study, but this number was intentionally chosen to allow for a detailed and controlled analysis of the involved variables. Previous studies on mathematical modeling in microcirculation started with small samples for initial model validation before being expanded to larger cohorts [25]. Even with a limited number of observations, these small samples carry relevant information about the system’s behavior, which allows for the identification of fundamental patterns and the validation of hypotheses before applying them to larger scales [25,26]. Our goal is to provide a proof of concept that can be expanded in future research.

## 2. Materials and Methods

The clinical trial was conducted by researchers A.K.A.d.S., J.C.T.-R. and S.d.S.R.F.R., who performed an analysis to be used in the computational simulations and the construction of the mathematical model. To ensure objectivity and evaluate the reliability of the model, this phase was conducted independently from the researchers of the initial clinical trial, basing the conclusions solely on the model’s performance and the clinical outcomes. The main hemodynamic parameters examined were capillary blood flow and pressure in the areas affected by the ulcers.

### 2.1. Study Design and Participants

From 2020 to 2023, a clinical study was conducted to address challenges faced by individuals with diabetic foot ulcers (DFUs). Ethical approval was obtained from the Federal University of Ceará’s Research Ethics Committee (Approval No. 4.470.914), and all participants provided written informed consent in compliance with Brazilian National Health Council Resolution No. 466/12. The study followed the ethical guidelines of the Declaration of Helsinki (1975). The study took place at the Integrated Center for Diabetes and Hypertension (CEADH) within a Primary Health Care Unit (UAPS) under the Unified Health System (SUS). Patients, who were referred for diabetes and hypertension complications, were selected from a randomized clinical trial: For this study, three DFU patients were randomly chosen and stratified based on ankle–brachial index (ABI) severity:

- Normal ABI (0.9–1.3);
- Moderate impairment (0.41–0.7);
- Severe impairment (≤0.4);
- Mockenberg calcification (≥1.4).

This stratification facilitated a focused analysis of ulcer severity and vascular complications, forming a basis for mathematical modeling and simulation. The small sample size was intentional, ensuring precise comparisons of vascular responses. Given the complexity of microvascular dynamics, a larger cohort would pose monitoring challenges. However, the collected data serve as a foundation for developing predictive models, which can later be refined and expanded for broader applications.

### 2.2. Literature Search

To obtain capillary parameters not clinically accessible, we conducted a literature search. We consulted PubMed, Web of Science, Scopus, IEEE Xplore, and Google Scholar, using terms such as “Artery”, “Capillary”, “Diameter”, “Wall”, “Blood”, “Pressure”, and “Viscosity”. We selected studies from the last 10 years involving healthy subjects, allowing for a comparison with our clinical data. This information validates and refines our in silico models, ensuring greater predictive accuracy.

### 2.3. Analysis of Clinical Data

**Figure 1:**
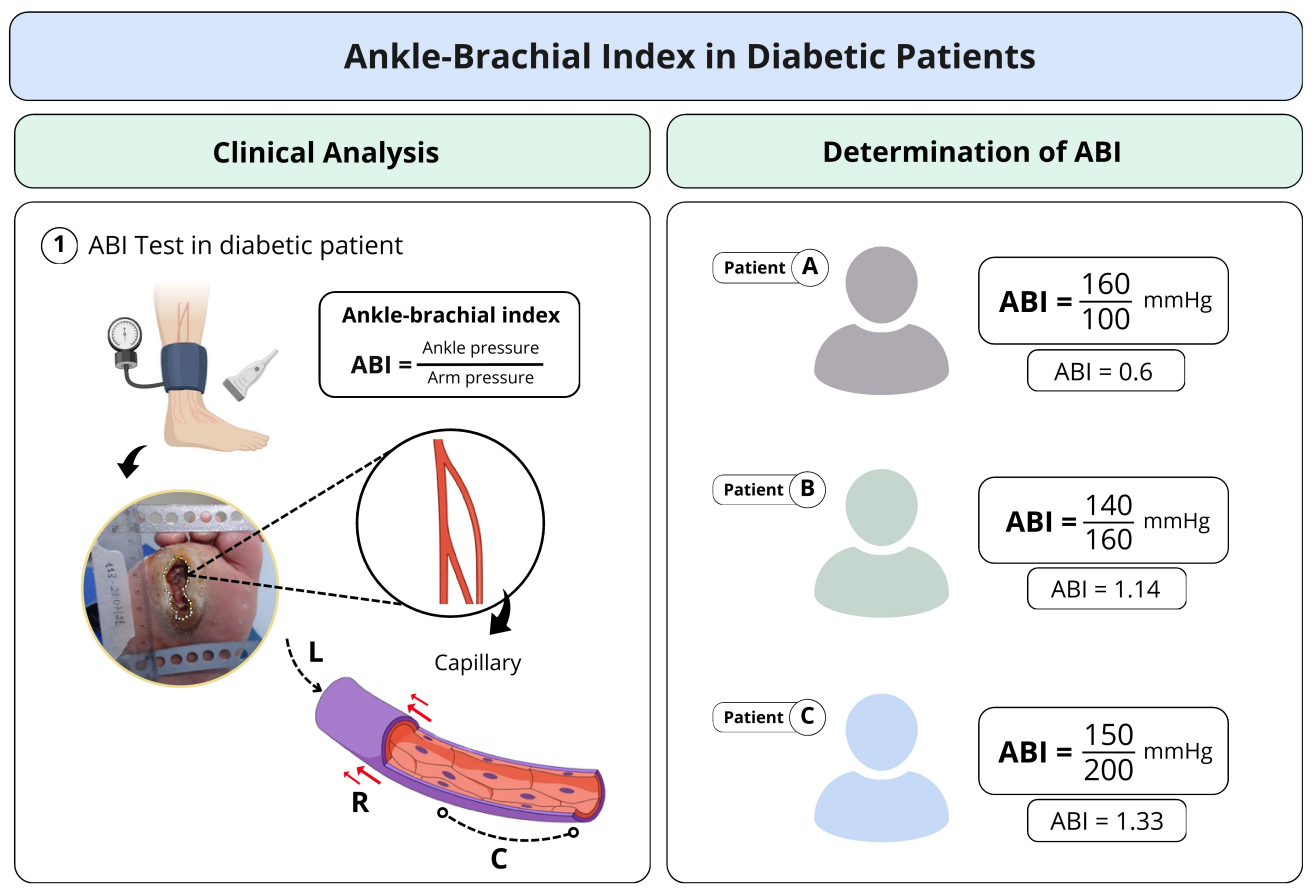
Representative scheme of the study methodology: the figure demonstrates the clinical collection stage of the ABI from diabetic patients presenting with diabetic foot wounds. The ABI, which provides blood pressure data, was calculated, and the other evaluated vessel variables served as the basis for analyzing arterial vessel responses in the parameters of the blood capillary.

The analysis of the clinical data in this study focused on the values of the ABI, photographs captured with a Sony Cyber-shot Silver DSC-W630 16MP digital camera mounted on a metal stand with a millimeter ruler positioned in the plane of the lesion, as well as the medical history and assessment of signs and symptoms of neuropathy, associated with vascular impairment. The ABI measurements were obtained using Doppler ultrasound, following the guidelines of the IWGDF. The values include measurements of both upper and lower limbs, analyzed individually as they may present variations (Figure 1). We considered the diastolic pressure of each limb, particularly in capillary analysis, rather than relying solely on the ABI calculation.

The small sample size allows for individualized analyses and detailed comparisons between different patient profiles, enabling the model to be adjusted and refined before being applied to a larger group. Future studies will include a greater number of participants to test the generalization of the findings.

### 2.4. BG Modeling

BG modeling is effective for systems that involve multiple physical phenomena, requiring large-scale consistency, modular structures, or reusable components. In this study, a capillary blood vessel model was used to address these criteria in the context of diabetes mellitus (DM). This approach helps to understand angiogenesis in chronic wounds and applies engineering principles to predict the system’s behavior under different conditions. The model, based on anatomical and physiological data, simulates capillary microvascular networks through the integration of biophysical principles of blood flow. A reductionist approach considered lower limb pressure to calculate the capillary variables. Represented as an equivalent electrical circuit with resistors and inductors, the model captures angiogenesis.

To validate the accuracy of the model, we compared the simulated parameters with literature data on microcirculation in diabetic patients [25]. The values of resistance and capacitance estimated by the model showed a correlation with those reported in previous studies, confirming the suitability of the mathematical approach used.

**Figure 2:**
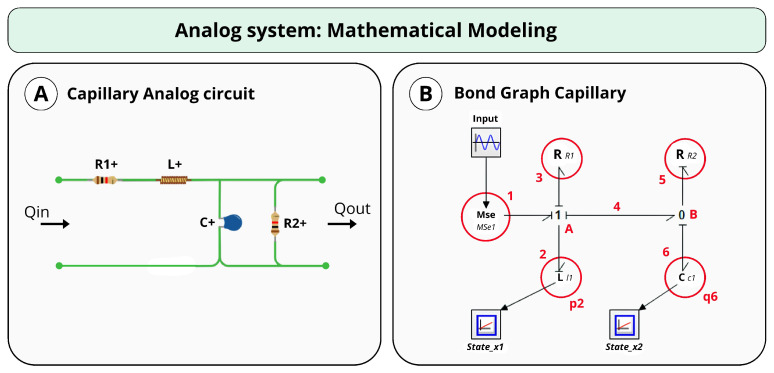
The analogous model represents the blood capillary through an equivalent electrical circuit. The resistor (*R*), capacitor (*C*), and inductor (*L*) correspond to the vessel’s resistance, compliance, and inertance, respectively. (**A**) The capillary analog circuit models blood flow, where $R_{1}$ and $R_{2}$ represent resistance, *C* represents compliance, and *L* accounts for inertance. (**B**) The bond graph representation illustrates energy flow within the system. The resistive (*R*), capacitive (*C*), and inductive (*I*) elements depict the vessel’s mechanical properties, while sources (*Se*, *Sf*) indicate external influences. The numbered nodes highlight key interaction points. Simulations using this model enable pressure and flow analysis in capillaries, aiding in the study of diabetic foot wounds and their progression.

In this scenario, the model was represented by an equivalent electrical circuit composed of a resistor $R_{1}$ and an inductor *L* in series, which are also in series with an equivalent impedance consisting of a resistor $R_{2}$ in parallel with a capacitor *C*, as illustrated in Figure 2. Table 1 shows the description of each component in the equivalent circuit.

**Figure 3:**
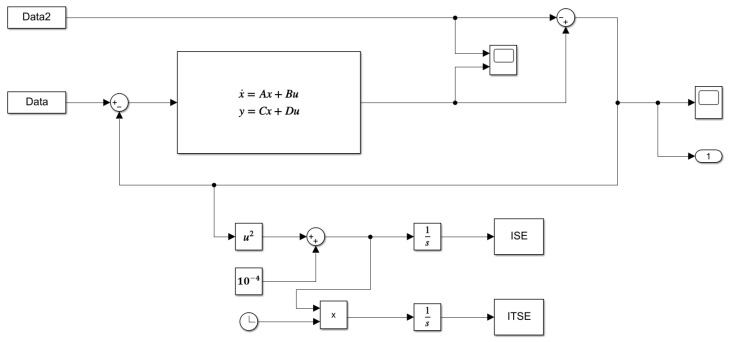
Block diagram of circulatory system executed in MATLAB Simulink^®^. Abbreviations are defined in the text.

Based on this model, the equivalent BG representation is shown in Figure 2 and Figure 3. Equations (1)–(11) depict the steps taken to achieve the final state-space model.

States:

(1) $$\dot{x}=\left[\begin{array}{cc} \dot{p_{2}} & \dot{Q_{6}} \end{array}\right]$$

Input:

(2) $$e_{1}=MSe$$

Applying the relationship between the elements,

(3) $$f_{2}=\frac{p_{2}}{L}$$

(4) $$e_{3}=R_{1}f_{3}$$

(5) $$f_{5}=\frac{e_{5}}{R_{2}}$$

(6) $$e_{6}=\frac{1}{C}Q_{6}$$

$$\begin{array}{cccc} A & \Leftrightarrow \mathrm{Junction}\mathrm{type}1 & B & \Leftrightarrow \mathrm{Junction}\mathrm{type}2\\ f_{1} & =f_{2}=f_{3}=f_{4} & f_{4} & =f_{5}+f_{6}\\ e_{1} & =e_{2}+e_{3}+e_{4} & e_{4} & =e_{5}=e_{6} \end{array}$$

For *L*, the system provides the effort to find state 1

(7) $$p_{2}=e_{2}=e_{2}-e_{3}-e_{4}\Rightarrow e_{2}=MSe-R_{1}f_{3}-\frac{1}{C}Q_{6}$$

(8) $$f_{3}=f_{2}\to e_{2}=MSe-\frac{R_{1}p_{2}}{L}-\frac{1}{C}Q_{6}$$

(9) $$\dot{x_{1}}=\dot{p_{2}}=-\frac{R_{1}}{L}p_{2}-\frac{1}{C}Q_{6}+MSe$$

For *C*, the system provides the flow

(10) $$\dot{x_{2}}=\dot{Q_{6}}=f_{6}=f_{4}-f_{5}=f_{2}-f_{5}=\frac{p_{2}}{L}-\frac{e_{5}}{R_{2}}$$

(11) $$\left\{\begin{array}{cc} \;e_{5}=e_{6}=\frac{1}{C}Q_{6} & \Rightarrow \dot{x_{2}}=\frac{1}{L}x_{1}-\frac{1}{R_{2}C}x_{2}\\ \dot{x_{2}}=\frac{p_{2}}{L}-\frac{1}{R_{2}C}Q_{6} \end{array}\right.$$

Finally, we find the structure of the state-space matrices, where the system ∈$R^{2}$ is given by Equations (12) and (13):

(12) $$\dot{x}\left(t\right)=Ax\left(t\right)+Bu\left(t\right)$$

(13) y(t)=Cx(t)+Du(t)

where matrix *A* (Equation (14)) is the state matrix, matrix *B* (Equation (15)) is the input matrix, the output matrix is represented by *C* (Equation (16)), and *D* is the transmission matrix, which is null.

(14) $$A=\left[\begin{array}{cc} -\frac{1}{R_{2}C} & \frac{1}{C}\\ -\frac{1}{L} & -\frac{R_{1}}{L} \end{array}\right]$$

(15) $$B^{T}=\left[\begin{array}{cc} \frac{1}{L} & 0 \end{array}\right]$$

(16) $$C=\left[\begin{array}{cc} 1 & 0 \end{array}\right]$$

The transfer function of the model, which relates the system’s output to the input, is given by $G\left(s\right)=\frac{Y(s)}{X(s)}$ (Equation (18)). From Equations (12) and (13), which show the classical representation of dynamic systems in state space, we obtain the transfer function using (17).

(17) $$\frac{Y(s)}{X(s)}=G\left(s\right)=\left[C{\left(sI-A\right)}^{-1}B\right]$$

(18) $$G\left(s\right)=\frac{\left|\begin{array}{cc} sI-A & -B\\ C_{j} & D_{ij} \end{array}\right|}{\left|\begin{array}{c} sI-A \end{array}\right|}$$

In the capillary modeling system, resistance is represented by *R*. We consider the total resistance $R_{t}$ as the sum of the individual resistance $R_{1}$, inductive reactance iωL and the impedance of a parallel circuit composed of a resistor $R_{2}$ and a capacitor *C*:

(19) $$R_{t}=R_{1}+i\omega L+\frac{R_{2}}{1+iR_{2}\omega C}$$

### 2.5. Determination of Parameters $R_{1}$, $R_{2}$, L and C and Transfer Functions

In this study, we simulated a 2 × 2 state-space linear system using the ODE23 solver (Bogacki-Shampine) on a computer equipped with a 2.2 GHz Intel^©^ Core I5-5200 (Intel Corporation, Santa Clara, CA, USA) processor and 8 GB of RAM, utilizing a student license of the software MATLAB^®^ R2021a (The MathWorks Inc., Natick, MA, USA).

To determine the parameters necessary for performing the dynamic analysis of the system, we used the block diagram shown in Figure 3. This diagram was designed in MATLAB–Simulink^®^, which operates by performing comparisons between the signal generated by the Data block, representing blood flow at the entry of the blood capillary, and the flow at the exit of the capillary (Data2), taking into consideration the various scenarios presented in this study. These datasets will be explored in Section 2.9.

The state-space block highlighted in Figure 3 contains all the matrices described in Equations (14)–(16), emphasizing the state matrix, which is composed of the circuit variables $R_{1}$, $R_{2}$, *L*, and *C*. In this work, we used performance indices based on the error integral criteria. We employed the integral of the squared error (ISE, Equation (20)) and the integral of the time-weighted squared error (ISTE, Equation (21)) [27,28,29].

These indices quantitatively describe the performance of the dynamic system for estimating the parameters of the analogous circuit. The integration limits are relative to the moments t=0 when the input signal represents the cardiac systole, which generates an increase in pressure in the capillary and consequently the flow, and *T*, which corresponds to the end of the cycle, i.e., the end of the respective diastole.

(20) $$ISE=\int_{0}^{T}e^{2}\left(t\right)dt\;,$$

(21) $$ISTE=\int_{0}^{T}te^{2}\left(t\right)dt\;,$$

In the ISE index, high values are obtained for significant errors and low magnitudes for small errors, thus reducing critical errors, especially at the initial moments. However, this index has low selectivity, which causes the system to exhibit a fast response but with low relative stability, inducing oscillations [29].

The ITSE index exhibits greater selectivity compared to the ISE criterion, operating in a weighted manner concerning the large initial errors with low weight. Near stabilization, this index severely penalizes errors in the transient response [29].

Simulink^®^ works in conjunction with a script developed in the MATLAB^®^ environment where the PSO algorithm was implemented. In this scenario, the objective function to be minimized is a linear combination of the described errors concerning the possible values of the components of the analogous circuit, as shown in Equation (22).

(22) argmin(OF=ISE+ITSE)

After the optimization process, we used the ss function to create a continuous-time state-space model. Subsequently, we obtained the open-loop transfer functions G(s) using the ss2tf. Additionally, we analyzed the characteristic polynomials using the Routh–Hurwitz (RH) stability criterion to evaluate stability based on the distribution of the system’s roots.

### 2.6. PSO Algorithm

This study utilizes PSO to adjust the critical parameters of a hemodynamic blood vessel model, aiming for an accurate representation of physiological behavior.

The PSO algorithm, introduced by Eberhart and Kennedy [30], draws inspiration from the collective behaviors of swarms, schools of fish, and bird flocks during their food search [31]. It employs computational search and optimization methods.

In PSO, each particle acts as a point mass in an N-dimensional space [32], where N corresponds to the number of adjustable parameters within the state-space block (Figure 3). Particles cooperate and compete to discover the optimal solution, with each particle representing a viable solution candidate [33,34]. They adjust their trajectory based on personal experience and shared information from other particles in the swarm. Particles are drawn toward the best solution identified by any member of their local neighborhood [29,35].

Initially, the swarm is randomly dispersed within the search space, characterized by position and velocity vectors [29,36]. The *i*th particle is thus defined by three vectors:

- The coordinates of an individual particle in the N-dimensional search space
  ${\vec{x}}_{i}=\left(x_{i,1},x_{i,2},\ldots ,x_{i,N}\right)$;
- The rate of change in position (velocity)
  ${\vec{v}}_{i}=\left(v_{i,1},v_{i,2},\ldots ,v_{i,N}\right)$;
- The position of best fitness that each particle has achieved (pbest)
  ${\vec{p}}_{i}=\left(p_{i,1},p_{i,2},\ldots ,p_{i,N}\right)$.

To achieve an optimal solution, PSO identifies the globally best position by evaluating the collective fitness of all particles. This position is denoted by gbest. The particles update their velocity and position by considering their current velocity, their distance from pbest, and their distance from gbest. When dealing with *S* particles, the position of the *i*th particle in the *j*th dimension is updated using Equations (23) and (24) [29].

(23) $$v^{k+1}i,j=\omega v^{k}i,j+c_{g}r_{1}\left(pbest_{i,j}-x^{k}i,j\right)+c_{s}r_{2}\left(gbest-x^{k}i,j\right)$$

(24) $$x^{k+1}i,j=x^{k}i,j+v_{i,j}^{k+1}$$

Here, *k* is the iteration index, and MaxIter is the maximum number of iterations, serving as a termination criterion. The algorithm operates while the condition 0<k≤MaxIter holds true.

The coefficients $c_{g}$ and $c_{s}$, representing cognitive and social components, respectively, affect the particle’s movement by balancing personal experience and swarm influence. These constants are generally fixed, though adaptive PSO variants can dynamically alter these values during optimization. The limits on maximum and minimum velocities ($V_{max}$ and $V_{min}$) and positions ($X_{max}$ and $X_{min}$) are predefined to ensure that particles remain within the search space [29].

Random numbers $r_{1}$ and $r_{2}$ are uniformly distributed between 0 and 1. The inertia weight ω moderates the velocity magnitude throughout the iterations. It is updated according to (25):

(25) $$\omega^{\left(k+1\right)}=\omega^{k}+\frac{\left(\omega_{f}-\omega_{0}\right)}{MaxIter}$$

where $\omega_{f}$ and $\omega_{0}$ are the final and initial inertia weights, which are typically set to 0.1 and 0.9, respectively [29].

Thus, the inertia weight is a scaling factor to modulate each particle’s velocity. Properly tuning ω can balance between global exploration and local exploitation, effectively minimizing the number of iterations needed to find an optimal solution [32].

### 2.7. Details of the MATLAB^®^’s Scripts

The implementation of PSO in MATLAB^®^ was organized into several scripts that work together to achieve the desired optimization.

The first script, named ParticleSwarmOptimization, is the main script that initializes the PSO algorithm and receives input parameters such as the number of particles (20), number of iterations (500), number of dimensions (4), maximum positions (450), and minimum positions (10^−6^), as well as the initial (0.9) and final (0.1) inertia factors. The initial velocity of the particles is calculated with twice the difference between the maximum and minimum positions. After this initialization, the Flow script is triggered.

The Flow script defines the input signals that simulate the behavior of flow and pressure in an arterial capillary. This script generates two datasets: one for normal blood flow and another for flows with different ankle–brachial index (ABI) values. Additionally, the Flow script plots graphs of the blood flow at the capillary’s entry and exit points.

The main script then calls the third script, called initialization. This script generates the random positions of the swarm particles, as well as the vector of the best individual positions. It also calculates the initial velocities and the initial value of the objective function. The values returned by initialization are used by the main script to proceed with the optimization process.

Within the main loop, which runs until the maximum number of iterations is reached, the main script determines the best position values and objective function using the PbestGbest script. This script receives information about the number of particles, objective function values from the previous iteration, particle positions, and the best global position up to that point. During this process, the script triggers a script called SSsimulation, which uses Simulink^®^ to perform simulations and calculate the updated objective function values.

Based on the position of the particles, Simulink^®^ calculates the objective function using ISE and ITSE metrics. The simulation results are returned to the PbestGbest script, which then determines the best individual and global positions of the particles.

The main script uses this information to update the velocities and positions of the particles, triggering the sixth script, called Update. This script updates the velocities and positions based on the PSO equations, taking into account social and cognitive factors, as well as the current inertia factor. If the particles exceed the search space boundaries, they are repositioned within those limits. The new velocities and positions are then returned to the main script for the next iteration. It is worth noting that the Update script performs a test to check whether the state-space system will diverge. This test is essential for the functioning of PSO within the Simulink^®^ environment to avoid simulation errors in the integrator blocks present in the ISE and ITSE errors.

After the loop reaches the maximum number of iterations, the final results are presented, including the optimized values of $R_{1}$, $R_{2}$, *L*, and *C*. These values are organized in a table, and the state-space structure is assembled to extract the transfer function that models the flow behavior within the blood vessel. The main script then calls the seventh script, called ControlPlot.

The seventh script, ControlPlot, is responsible for plotting the graphs related to the simulation, including the response to a unit step, a unit impulse, and the convergence of the PSO algorithm over the iterations. Additionally, this script graphically shows the response of the dynamic system with the application of the input signal representing blood flow at the capillary entrance during systole.

In addition to the main PSO scripts, there is a separate script called VaryGraphs, which applies the values of $R_{1}$, $R_{2}$, *L*, and *C* to step functions to vary these parameters between 0 and 0.5, allowing observation of how each variable affects the system’s behavior. Additionally, the source code folder for simulations, available in the Supplementary Materials, contains saved simulations that depict tests conducted during the development of the code. Finally, a last script, called manual, reproduces the results presented in this study.

This detailed description of the operation of the MATLAB^®^ scripts, along with the corresponding flowchart, provides a comprehensive overview of how the PSO algorithm is implemented and applied for parameter optimization in dynamic systems simulated in Simulink^®^.

In summary, the steps for determining the parameters of matrix A (Equation (14)) are shown below.

1. Initialization: The particles (possible solutions) were initialized with random values for the parameters $R_{1}$, $R_{2}$, *L*, and *C*.
2. Evaluation: Each particle was evaluated based on the objective function (22), which measures the difference between the model output and the real hemodynamic data.
3. Update: The particles were updated based on their own best-known positions and the best-known positions of the swarm.
4. Convergence: The process continued iteratively until a stopping condition was met, such as a maximum number of iterations or convergence in parameter values.

### 2.8. PSO Validation

The validation of the PSO algorithm using another state-space system is essential to ensure the robustness and reliability of the method in determining the parameters *R*, *L*, and *C*. To achieve this, testing the PSO on a model where the inputs, outputs, and optimal parameter values are already known allows for a direct comparison between the results obtained by the algorithm and the reference values. This approach enables the evaluation of PSO’s ability to find solutions close to or identical with the expected values, as well as its efficiency in terms of convergence and stability.

Furthermore, validation with another system reduces the uncertainty associated with the method and ensures that it is not just fitting the parameters for a specific case, but rather offering a generic solution applicable to different dynamic models. This step is crucial to ensure that the PSO can be used with confidence in the optimization of complex systems, guaranteeing that the obtained responses are realistic and effective for real-world applications.

**Figure 4:**
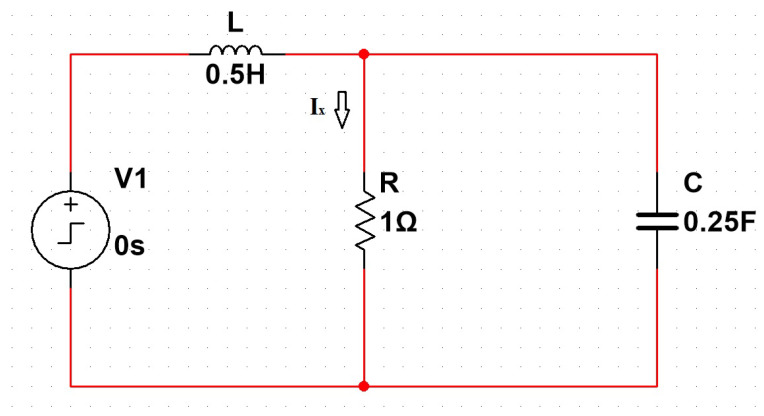
Validation system. Adapted from [37].

In this scenario, we conducted simulations on the system shown in Figure 4. In contrast to the circuit discussed in this study (Figure 2), this system features only one resistor while maintaining the same state-space structure. This approach allows for the evaluation of the PSO algorithm’s effectiveness in accurately determining the model parameters, ensuring that the methodology is applicable to different dynamic system configurations.

The system ∈$R^{2}$ is represented by the state-space structure in Equations (26) and (27).

(26) $$\left[\begin{array}{c} \dot{v}\\ \dot{i} \end{array}\right]=\left[\begin{array}{cc} -\frac{-1}{RC} & \frac{1}{C}\\ -\frac{-1}{L} & 0 \end{array}\right]\left[\begin{array}{c} v\\ i \end{array}\right]+\left[\begin{array}{c} 0\\ \frac{1}{L} \end{array}\right]v_{s}$$

(27) $$i_{x}=\left[\begin{array}{cc} \frac{1}{R} & 0 \end{array}\right]\left[\begin{array}{c} v\\ i \end{array}\right]$$

### 2.9. Generation of the System’s Input and Output Signal

For the blood flow simulation, we created an input signal as modeled by Equation (28), representing 72 beats per minute (BPM) with amplitudes extracted from Table 2 [38]. This signal has a period of $\frac{60}{72}$ s, of which $\frac{2}{5}$ represents the systole phase. Additionally, we created a second signal, phase-shifted by 90 degrees, with an amplitude equal to the flow calculated from the ABI measurements of the study’s participants, based on Poiseuille’s law (see Equations (29) and (30) and Table 3) [39]. This process was conducted to represent the blood flow in the capillary of an individual affected by diabetes (output of the equivalent electrical circuit represented in the BG model).

(28) $$Q\left(t\right)=\left\{\begin{array}{cc} \;Q_{0}\times sin^{2}\left(\frac{\pi \;\cdot \;t}{T_{s}}\right) & t\in (0,T_{s})\\ 0 & t\in (T_{s},T) \end{array}\right.$$

(29) $$Q=\frac{\Delta p}{R}$$

(30) $$R=\frac{8\eta l}{\pi r^{4}}$$

where

- $Q_{0}$ is the peak flow determined by the calculations derived from the ABI.
- *T* is the Period.
- $T_{H}$ is the period that covers the time interval of cardiac systole and diastole.
- $T_{s}=\left(\frac{2}{5}\cdot T_{H}\right)$ Time in systole.
- Δp is the pressure variation.
- *R* is the resistance.
- *l* is the blood capillary length.
- *r* is the blood capillary radius.
- η is the viscosity of the blood capillary.

## 3. Results

This study employed capillary modeling in diabetic wounds using the BG method, integrating principles from engineering and health to enhance the understanding of these wounds. Based on our literature review, we did not find any similar studies that use accurate patient data with diabetic ulcers, highlighting the novelty of our approach. The ABI was a crucial variable in the assessment, providing a practical correlation between theoretical modeling and the clinical reality of patients. Additionally, the PSO algorithm is used to optimize the model parameters. The inclusion of clinical data aimed to validate the applicability and accuracy of the mathematical modeling, emphasizing the clinical relevance and potential effectiveness of combining engineering approaches with medical metrics.

Despite the increasing availability of advanced diagnostic methods, the ABI remains, after more than seven decades of use, a fundamental method for the non-invasive assessment of patients with PAD, both symptomatic and asymptomatic. It serves as a crucial epidemiological tool, allowing for the mapping of PAD progression and assessing cardiovascular risk [48]. It is effective in identifying individuals with developing atherosclerosis who require stringent preventive measures [49].

The samples utilized to analyze the proposed mathematical model exhibited a range of ankle–brachial index (ABI) values, including those within the normal range (ABI = 1.14), below the normal range (ABI = 0.6), and above the physiological range (ABI = 1.33). In addition to examining these ABI values, this study explored the hemodynamic factors associated with systolic pressure in each lower limb presenting with ulcers. This multifaceted approach facilitated a deeper understanding of the patients’ vascular conditions by correlating systolic pressure levels with the severity and progression of diabetic ulcers. The results obtained are detailed below, emphasizing the relationship between systolic pressure and the observed clinical outcomes. Furthermore, crucial data for the simulation and modeling were gathered through a comprehensive literature search, which provided a robust foundation for the development of the mathematical model and ensured that the parameters employed were both relevant and reflective of real-world conditions.

We performed sensitivity analyses to assess the impact of variations in the parameters on the obtained results. These analyses indicated that small changes in the values of peripheral resistance and capacitance significantly alter the flow patterns, which is consistent with experimental studies on the hemodynamics of patients with diabetic ulcers [7].

### 3.1. Physiological Parameters of Capillaries Found in the Literature

From the literature search, ten articles were included in this study to compile a table of physiological parameters of the physical, hemodynamic, and mechanical properties, including diameter, wall thickness, blood viscosity, internal pressure, and density. These variables were crucial for validating our hemodynamic model, allowing for a comparison with a sample without vascular impairments, as with the patients evaluated in this study.

An ABI of 1 was used as a reference value in our study to standardize and compare the physical and physiological parameters of arterial and capillary vessels. An ABI of 1 indicates an average ratio between ankle blood pressure and arm blood pressure, serving as a benchmark for assessing vascular health.

The articles only contained flow and resistance parameters, which were found through formulas (Equations (29) and (30)). Flow rate measures how much blood passes through a vessel in a specific amount of time. In our study, we calculated the flow rates for arterial and capillary vessels based on the provided dimensions and physiological parameters. Vascular resistance indicates the amount of opposition the blood encounters as it flows through the vessels. It is determined by the physical dimensions and hemodynamic properties of the vessels. Higher resistance values in the capillaries compared to the arteries reflect their smaller diameter and the resulting opposition to blood flow [39].

### 3.2. Data and Modeling of ABI

The patients’ evaluation records were consulted, including the values of blood pressures from the upper and lower limbs and the calculation of the ABI. These values were transcribed into Table 4 and served as the basis for the proposed equations and mathematical model.

In this study, we prioritized lower limb pressure measurements to calculate the capillary models’ scaling factors. The most relevant parameter was the pressure in the posterior tibial artery, which branches into the capillaries being analyzed.

The global ABI provides an overall average of arterial pressures between the ankle and the arm, combining measurements from different parts of the body. However, to accurately model capillary dynamics, it is important to consider the specific pressures in the lower limbs where the capillaries are located. The global ABI may not adequately capture regional variations that are critical for understanding capillary dynamics.

By focusing on specific measurements for the lower limbs, we were able to simplify the model while maintaining its accuracy and relevance for the specific vascular conditions analyzed. The parameters for the arteries and capillaries were defined based on standard values from the literature, as described in Table 2. Meanwhile, we calculated the capillaries using the ABI, which was the main focus of this study.

### 3.3. Clinical Case Analysis: Patient A

**Figure 5:**
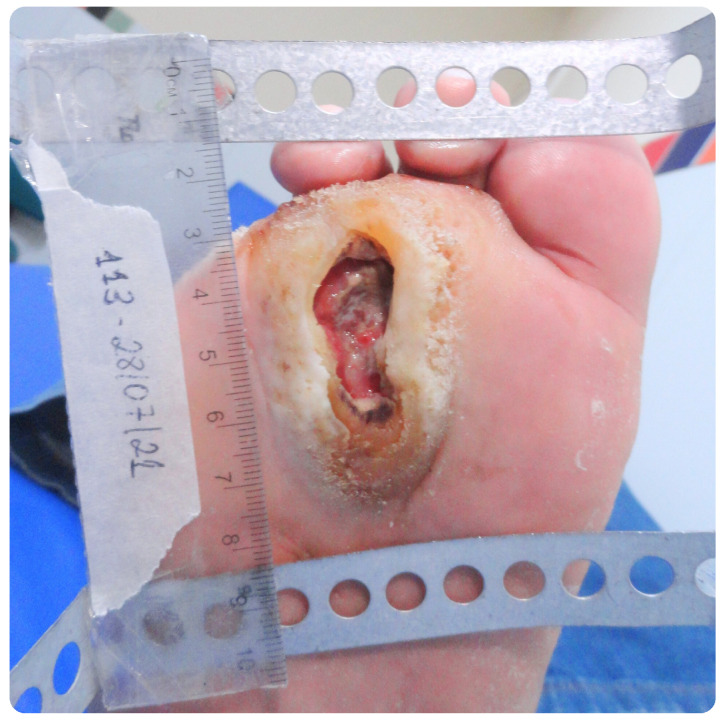
Image of the deep ulcer on the plantar region of the right foot of Patient A. The ulcer extends to the osteoarticular tissues and has persisted for over a year without signs of healing, despite conventional treatments. Clinical measurements include: systolic blood pressure in the lower limbs—160 mmHg; ABI for both lower limbs—1.00; ipsilateral ABI—0.6. The wound size measures 3.978 cm^2^.

Patient A, a 66-year-old male, has been diagnosed with type 2 diabetes mellitus (DM2) for approximately 33 years, with blood glucose levels controlled by medication. He is also hypertensive and has peripheral neuropathy. He has a history of amputations of the fourth and fifth toes on their right lower limb. He presented to the healthcare service reporting a deep ulcer on the plantar region of their right lower limb involving osteoarticular tissues, as shown in Figure 5. On the neuropathic impairment scale, he exhibited moderate symptoms. The ulcer had been present for over a year, and despite conventional treatments at a unit within their health plan, it showed no signs of healing. The ABI of 0.6 on the side of the wound clinically suggests moderate to severe PAD.

### 3.4. Clinical Case Analysis: Patient B

**Figure 6:**
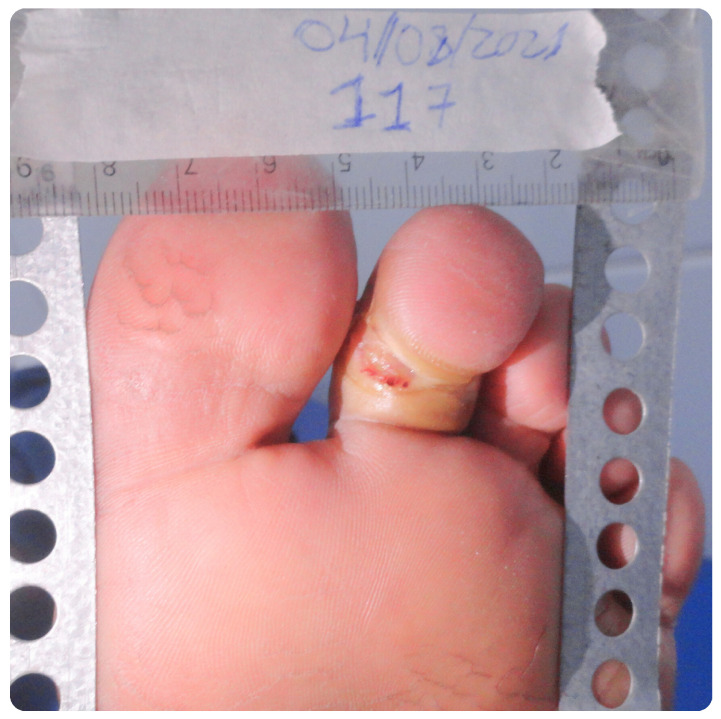
Image of the ulcer on the plantar region of the left foot of Patient B. Clinical measurements include: systolic blood pressure in the lower limbs—140 mmHg; ABI for both lower limbs—1.60; ipsilateral ABI—1.14. The wound size measures 0.357 cm^2^.

Patient B, a 67-year-old male, has been diagnosed with type 2 diabetes mellitus for 30 years. Despite medication, their glycemic levels are poorly controlled. He also has a history of hypertension, dyslipidemia, and prostate cancer, treated six years ago with medical clearance. He presented with moderate signs and symptoms of neuropathic impairment. He reported a wound on the plantar region of the second toe of their left foot, which appeared after stepping on a nail about 6 months ago and had not healed, as shown in Figure 6. The ABI showed values within the normal range.

### 3.5. Clinical Case Analysis: Patient C

**Figure 7:**
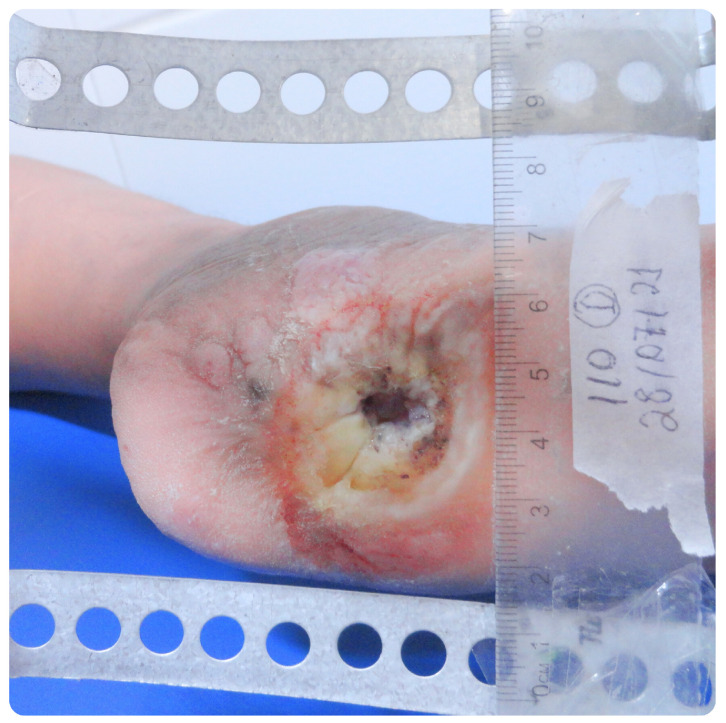
Image of the ulcer on the plantar region of the right foot of Patient C. Clinical measurements include: systolic blood pressure in the lower limbs—150 mmHg; ABI for both lower limbs—2.00; ipsilateral ABI—1.33. The wound size measures 6.038 cm^2^.

Patient C, a 47-year-old female, has been diagnosed with DM2 for 15 years. She also has diabetic retinopathy, dyslipidemia, and diabetic nephropathy, which led to a left nephrectomy with no history of amputations. She has a history of ulcers on the contralateral lower limb. The neuropathic symptoms score indicated severe neuropathy. At the time of evaluation, she had an ulcer on the heel of her right lower limb, as shown in Figure 7. The ulcer had been present for about three years and, despite treatment, did not heal and frequently became infected. With an ABI of 1.33, it can be considered that the patient did not have detectable ischemia based on this specific value.

### 3.6. Parameters Determined by the PSO

The proposed model utilizes electrical circuit components to represent the hemodynamic behavior of capillaries, offering an innovative approach to simulating blood flow dynamics at the capillary level in the context of DM. In this study, a single transfer function was derived from the state-space representation generated by the BG model.

**Figure 8:**
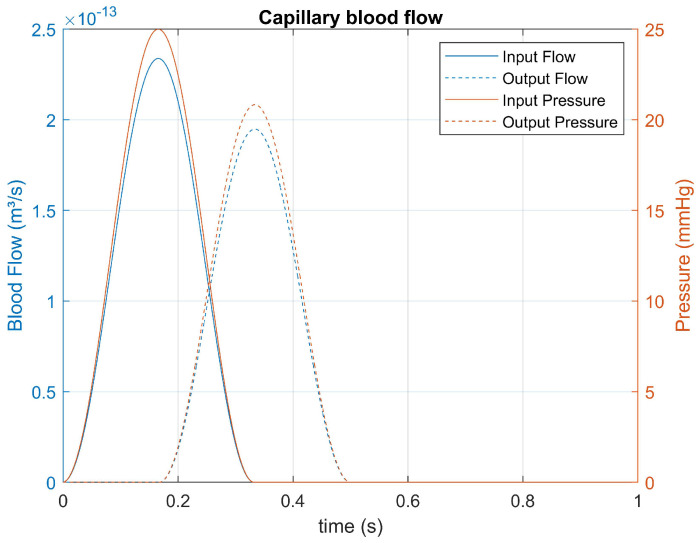
Signals applied to the electrical circuit analogous to the blood capillary.

The input and output blood flow signals discussed in Section 2.9 are presented in Figure 8.

With the application of the PSO algorithm, the parameters $R_{1}$, $R_{2}$, *L*, and *C* were determined (Table 5), resulting in the transfer function presented in Equation (31).

**Figure 9:**
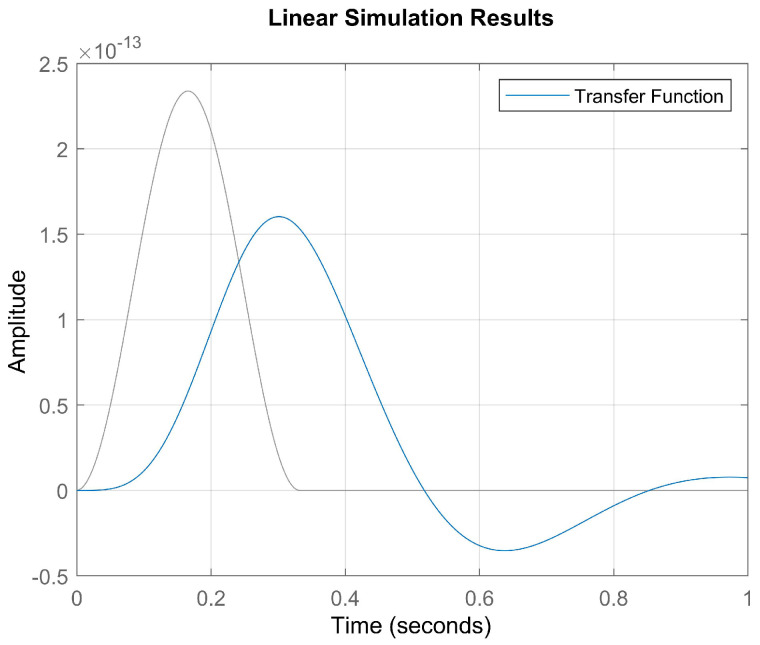
Simulation of the system’s response. After determining the parameters $R_{1}$, $R_{2}$, *L*, and *C*, as well as deriving the transfer function from the state-space equations, the same input flow (Figure 8) from the equivalent circuit was applied to the model (gray line). The results show that the simulated output closely matches the expected output signal, validating the accuracy of the obtained mathematical model and its capability to faithfully represent the dynamics of capillary blood flow.

After determining the parameters, the input signal shown in Figure 8 was applied to the transfer function, yielding the result presented in Figure 9.

(31) $$G\left(s\right)=\frac{89.38}{s^{2}+9.028s+108.2}$$

**Figure 10:**
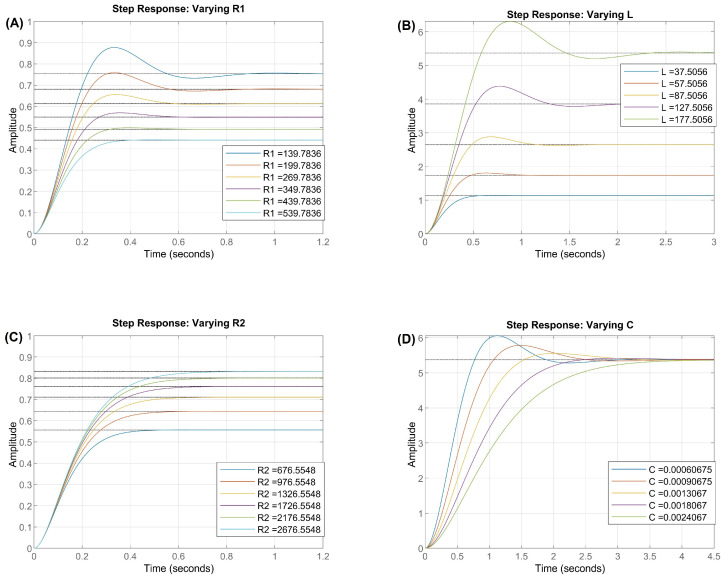
Step responses of the circuit for different parameter variations, demonstrating the influence of electrical components on the system’s dynamic behavior. The increase in primary resistance, $R_{1}$ (**A**), leads to higher damping, reducing the peak amplitude and minimizing oscillations. The inductance variation, *L* (**B**), affects the response time, where higher values delay stabilization and may induce underdamped oscillations. The impact of secondary resistance, $R_{2}$ (**C**), is observed in the attenuation of oscillations and faster convergence to steady-state conditions as its value increases. Finally, an increase in capacitance, *C* (**D**), results in a slower response with reduced overshoot, whereas lower values lead to faster but more oscillatory behavior. These results highlight the importance of appropriately selecting circuit parameters to optimize the system’s transient response.

The conducted experiments demonstrate the influence of circuit parameters on the step response, highlighting the impact of resistance, inductance, and capacitance on the dynamic behavior of the system. The analysis revealed that increasing the primary resistance, $R_{1}$ (Figure 10A), leads to more pronounced damping, reducing the maximum peak of the response and minimizing oscillations. This behavior suggests a more efficient dissipation of the energy stored in the system, resulting in a more stable and less oscillatory response.

The variation in inductance (Figure 10B) showed a significant effect on the system’s response time. Higher values of *L* resulted in a greater delay in the stabilization of the response, as inductance acts as an element that restricts rapid changes in current. Additionally, it was observed that for certain values of *L*, the system’s response exhibited more intense oscillations, indicating an underdamped behavior.

The influence of the secondary resistance, $R_{2}$, (Figure 10C) was characterized by a reduction in the amplitude of oscillations as its values increased. The energy dissipation provided by this resistance contributed to more efficient damping, decreasing the time required to reach steady-state conditions. This behavior reinforces the role of $R_{2}$ in controlling the system’s stability, making it a relevant factor in determining the transient response.

Finally, the analysis of capacitance (Figure 10D) indicated that an increase in this parameter resulted in a slower response, but with a lower overshoot. Capacitors with higher values store more charge, delaying the voltage stabilization and increasing the time required to reach the steady state. On the other hand, smaller values of *C* favored a faster response but with more pronounced oscillations, highlighting the influence of capacitance on the system’s natural frequency.

### 3.7. Validation Results

To validate the effectiveness of the PSO algorithm in determining the model parameters in state space, two validation experiments were conducted using test circuits with known exact values: R=1, L=0.5, and C=0.25.

**Figure 11:**
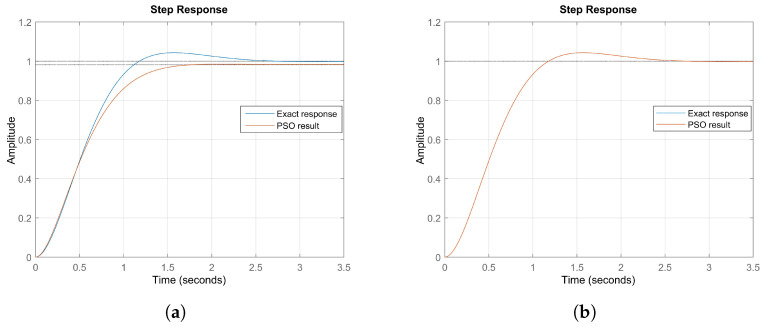
Unit step response validation of the system using the PSO algorithm. (**a**) In the first validation experiment, the estimated parameters, R=1.0187, L=0.57453, and C=0.17955, resulted in a response closely matching the actual system. (**b**) In the second experiment, the optimized parameters R=1.0889, L=0.42664, and C=0.22409 achieved an even more precise fit, with the simulated response almost perfectly overlapping the real system’s response, demonstrating the high accuracy of the PSO-based parameter estimation. The dotted line marks the final value, indicating the system’s stabilization.

In the first experiment, PSO was applied to estimate these parameters based on the system’s input and output data, resulting in the following values: R=1.0187, L=0.57453, and C=0.17955. Figure 11a presents the unit step response of the system, comparing the actual system with the response generated by the model adjusted by PSO. The results indicate that the obtained values are close to the expected ones, demonstrating the algorithm’s ability to accurately identify the system parameters and reinforcing its applicability in modeling dynamic systems with a state-space representation.

In the second validation experiment, a new optimization run was performed, yielding even more precise parameter estimates: R=1.0889, L=0.42664, and C=0.22409. The unit step response, shown in Figure 11b, exhibits an almost perfect overlap between the simulated and actual system responses, further confirming the high accuracy of the PSO algorithm in determining the optimal parameters.

### 3.8. Dynamic Analysis of the Analog Circuit Originating from the BG Model

The analysis of the dynamic response of the system modeled through the transfer function obtained by the PSO algorithm provides a deeper understanding of the effects of physiological properties on blood flow in the capillary. The transfer function presents complex conjugate poles at s=−4.5139±9.3713i, indicating that the system exhibits an underdamped behavior, characterized by oscillations in the transient response before stabilizing at the steady state. This behavior is crucial in the context of hemodynamics, as it reflects the interactions between vascular resistance, capillary elasticity, and variations in blood pressure.

**Figure 12:**
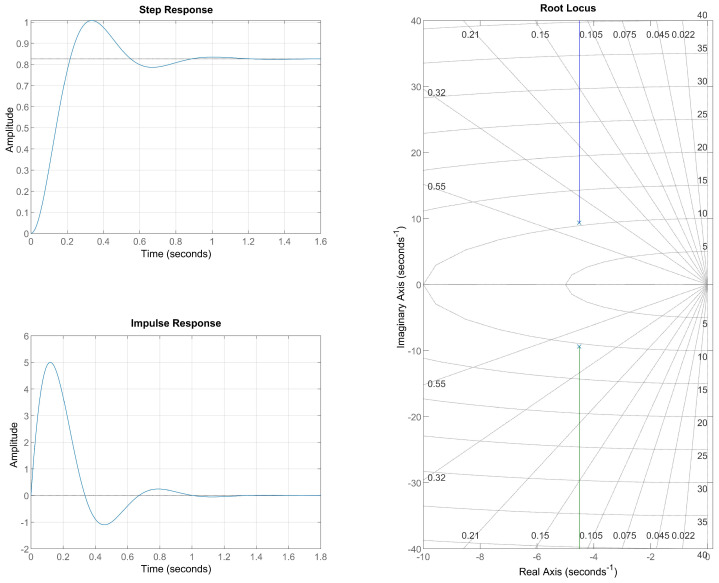
Dynamic responses of the modeled system for capillary blood flow. The graph on the left above presents the unit step response, highlighting transient oscillations before the flow stabilizes, characteristic of an underdamped system. In the left below, the unit impulse response demonstrates how the flow reacts to sudden pressure changes, displaying oscillations before reaching a steady state. On the right, the root locus diagram shows the position of the poles in the left half-plane of the complex plane, confirming the system’s stability but indicating the presence of transient oscillations due to the proximity of the poles to the imaginary axis. The dotted line on the step and impulse responses marks the final value, indicating the system’s stabilization. The transfer function presents complex conjugate poles at s=−4.5139±9.3713i. In the root locus diagram, the green and blue lines represent the asymptotes of the pole trajectories, indicating the direction the poles follow as the gain *K* varies from 0 to infinity. These lines serve as visual tools to understand the movement of the system’s poles and to predict stability variations as the gain changes.

Figure 12 shows the unit step response, the unit impulse response, and the root locus of the obtained system. The presence of complex poles suggests that the dynamics of blood flow exhibit controlled oscillations, which may be related to the resilience of the microcirculation in maintaining an efficient distribution of blood in the tissues. The relative damping parameter of these poles directly influences the settling time of the capillary flow, as well as the intensity of the observed oscillations.

In the context of diabetes mellitus DM, these characteristics become even more relevant. In healthy individuals, capillary microcirculation is highly adaptive, allowing dynamic adjustments in blood flow according to the metabolic needs of the tissues. However, diabetic patients often experience impairments in vascular elasticity and in the autoregulation of blood flow, which can lead to abnormal oscillations or instabilities in blood supply. The analysis of the obtained transfer function suggests that the dynamics of capillary circulation may be influenced by factors such as increased blood viscosity and reduced vascular compliance, phenomena commonly found in the pathophysiology of diabetes.

The system’s unit step response shows that, despite the initial oscillations, blood flow eventually stabilizes. The presence of these transient oscillations may indicate a slower system response to changes in blood pressure, a concerning factor for patients with diabetes mellitus. Excessive oscillations in blood flow can impair tissue perfusion and increase the risk of microvascular complications, such as diabetic retinopathy.

The unit impulse response provides further insight into the system’s ability to handle sudden disturbances in blood flow. As expected for an underdamped system, the impulse response exhibits oscillations before returning to the baseline state. This suggests that, in a physiological scenario, blood flow may exhibit temporary variations following sudden stimuli, such as a rapid change in blood pressure. In a healthy system, these oscillations are quickly dissipated, allowing for efficient autoregulation of the microcirculation. However, in diabetic patients, capillary stiffness may lead to prolonged oscillations, making it more difficult for the blood flow to adapt to changes in hemodynamic conditions.

The root locus analysis shows that the poles of the system are positioned in the left half-plane of the complex plane, ensuring the system’s stability. However, the proximity of the poles to the imaginary axis indicates that the oscillations are relatively persistent before the flow reaches a steady state. This factor may be associated with difficulties in regulating capillary pressure in diabetic patients, increasing susceptibility to endothelial damage due to cyclical fluctuations in blood flow.

The combined interpretation of the step and impulse responses confirms that the model realistically reflects the dynamics of blood flow in capillaries, especially in the context of diabetic patients. The observed oscillations suggest a significant interaction between vascular resistance and capillary elasticity, directly influencing the adaptation of blood flow to variations in blood pressure. The overall stability of the system, despite the presence of transient oscillations, indicates that capillary circulation maintains an adequate level of control over the flow. However, potential dysfunctions in autoregulation could compromise the efficient distribution of blood in the tissues.

These findings are crucial for validating the model as a reliable tool for analyzing capillary hemodynamics, enabling future investigations aimed at optimizing therapeutic strategies for circulatory disorders associated with diabetes mellitus DM.

### 3.9. Data from the Modeling of Pressure x Flow Parameters in Capillaries

Simulations were performed by inputting the state-space equations into MATLAB, allowing the PSO algorithm to determine the parameters $R_{1}$, $R_{2}$, *L*, and *C*. The ABIs were collected from the patients. The pressures were calculated based on the systolic pressure values of the posterior tibial arteries.

A comparative analysis between the average physiological data from the literature and the values obtained for our patients revealed significant insights. By keeping the parameters of diameter, radius, length, and thickness of the arteries and capillaries constant, variations in flow and pressure in the capillaries among the three participants were observed.

Patient A had an initial arterial pressure measured at 100 mmHg, below the average of 120 mmHg, suggesting possible peripheral hypotension. The capillary pressure was also below the expected average of 25 mmHg, with a value of 20.83 mmHg. Although Patient A’s arterial flow of $4.42\times 10^{-4}\;m^{3}/s$ and capillary flow of $1.95\times 10^{-13}\;m^{3}/s$ were within normal ranges, the capillary resistance was notably high, reaching $1.43\times 10^{16}\;\mathrm{Pa}\cdot s/m^{3}$. This value is significantly higher than the average in the literature, indicating a markedly increased capillary resistance that could impact microcirculation.

Patient B had an initial arterial pressure of around 160 mmHg, indicating peripheral hypertension, which is significantly above the average of 120 mmHg. Consequently, the capillary pressure was also above average, with 33.33 mmHg compared to 25 mmHg. The arterial flow, measured at $7.07\times 10^{-4}\;m^{3}/s$, was slightly higher than expected, while the capillary flow of $3.12\times 10^{-13}\;m^{3}/s$ was within the normal range. The capillary resistance was also high, with a value of $1.43\times 10^{16}\;\mathrm{Pa}\cdot s/m^{3}$, reflecting increased resistance similar to that observed in Patient A.

Patient C had an initial arterial pressure of 200 mmHg, which is well above the average of 120 mmHg and indicates severe hypertension. The capillary pressure was significantly higher than average, with a value of 41.67 mmHg compared to 25 mmHg. The arterial flow of Patient C, at $8.83\times 10^{-4}\;m^{3}/s$, and the capillary flow of $3.90\times 10^{-13}\;m^{3}/s$ were in line with expectations. The observed capillary resistance, at $1.43\times 10^{16}\;\mathrm{Pa}\cdot s/m^{3}$, remained consistently high, similar to that of the other patients.

These findings highlight the importance of considering arterial pressure and capillary resistance in diagnosing and treating vascular conditions. The mathematical modeling used in this study proved to be a promising tool for identifying and analyzing these variations, offering a deeper understanding of capillary hemodynamics and their clinical implications.

## 4. Discussion

The analysis of patient data, including ulcer size, onset time, and the ABI, revealed a notable interaction between the severity of ischemia and wound healing in our sample. For instance, Patient A, with a 3.978 cm^2^ ulcer that appeared over a year ago, had an ABI of 0.6, indicating moderate to severe ischemia. This low ABI suggests compromised peripheral circulation, contributing to the wound’s large size and prolonged duration, likely increasing the risk of infection. The persistence of this ulcer underscores the severe vascular insufficiency that impairs healing.

Patient B, in contrast, has a smaller ulcer of 0.357 cm^2^, which appeared after trauma about six months ago. His normal ABI of 1.14 suggests no detectable ischemia, consistent with the recent onset and smaller size of the ulcer. However, the absence of ischemia does not rule out other factors influencing healing, such as peripheral hypertension or poor glycemic control. Non-healing in this case may point to complications beyond ischemia, like infection or improper treatment.

Patient C presents with the largest ulcer (6.038 cm^2^), which developed approximately three years ago. Despite an elevated ABI of 1.33, which generally indicates no ischemia, the severe hypertension observed may hinder perfusion and healing, highlighting how hypertension can impair wound recovery, even in the absence of ischemia. High blood pressure reduces microcirculation efficiency, aggravating inflammation and tissue damage, which contributes to the persistent nature of the ulcer.

Our findings align with the results of [50], who identified a strong correlation between ABI values and wound healing in diabetic foot ulcers. In particular, patients with ABI values below 0.6, like Patient A, had more severe and harder-to-heal ulcers due to compromised peripheral circulation. Similarly, patients with normal ABI values, like Patient B, although free of ischemia, faced healing challenges related to factors like infection or inadequate glycemic control. In patients with an elevated ABI, such as Patient C, conditions like hypertension were shown to negatively impact wound healing despite the absence of ischemia. These results underscore the multifactorial nature of diabetic ulcer healing, emphasizing the roles of the ABI, hypertension, and glycemic control in determining recovery outcomes [50].

Furthermore, a study of patients over 45 years undergoing foot and ankle surgery highlighted the significant prevalence of peripheral artery disease (PAD) in those with impaired wound healing. This finding reinforces the importance of preoperative ABI screening, which could help identify patients at higher risk for delayed wound healing post-surgery [51].

A total of 194 patients with diabetic foot ulcers (DFUs) hospitalized between January 2009 and January 2011 were analyzed, with a one-year follow-up. The patients were divided into three groups based on prognosis: amputation, non-healing, and healed. Factors such as age, gender, educational level, family history of diabetes, and ulcer severity (classified by the Wagner system) were evaluated. The study observed that the ankle–brachial index (ABI) was an important factor in the prognosis of diabetic foot ulcers. Patients in the amputation group had significantly lower ABI values compared to the other groups. Multiple regression analysis indicated that the ABI was positively correlated with ulcer recovery, suggesting that higher ABI values are associated with better outcomes in DFU treatment. The ABI, which assesses the presence of peripheral arterial disease, reflects blood circulation in the lower limbs and plays a crucial role in evaluating ulcer severity and guiding clinical interventions [52].

### Engineering Aspects in Capillary Simulations

Due to the complexity of the vascularization of a diabetic foot in terms of anatomy and function, there are difficulties in performing in vivo measurements across the full range of physiological stresses that occur and can lead to poor wound healing. Therefore, simulations provide a numerical solution to differential equations to predict biological behavior [53].

The arterial parameters were defined based on standard values from the literature, as described in Table 2. Meanwhile, the capillaries were calculated using the ankle–brachial index (ABI), which was the main focus of this study.

The initial arterial pressure was observed in Patients A to C (100 mmHg to 200 mmHg). The flow in the arteries also increased proportionally. In contrast, the flow in the capillaries was shallow and exhibited only slight variations. The resistance in the arteries was constant among patients, indicating a high and uniform resistance to blood flow. The resistance in the capillaries was considerably higher due to the smaller diameter and cross-sectional area.

Capillary resistance in diabetes can vary due to a balance between factors that increase resistance, such as basal membrane thickening and glycation, and factors that decrease resistance, such as capillary dilation and the formation of new vessels. This dynamic behavior reflects the complexity of the vascular system in response to diabetic stress and can lead to variability in capillary resistance depending on the stage and severity of the disease, as well as the compensatory strategies of the body.

Prolonged glucose elevation in diabetes can significantly damage the endothelial cells lining the capillaries, resulting in oxidative stress, inflammation, and endothelial dysfunction. These factors compromise capillary function, increasing capillary resistance and reducing the efficiency of gas and nutrient exchange. Decreased tissue perfusion is particularly critical in areas such as the feet and legs, where proper circulation is essential for wound healing and maintaining tissue health.

In response to decreased perfusion and chronic stress, the body may attempt to compensate through angiogenesis, forming new blood vessels from existing ones. However, in diabetes, angiogenesis may be inadequate or dysfunctional. The newly formed vessels may not be fully functional due to the ongoing presence of glycation and basal membrane thickening, factors that exacerbate capillary resistance.

When capillary resistance remains high, even angiogenesis may not restore adequate perfusion. The newly formed capillaries may exhibit constant resistance along their length and throughout the blood pulse cycle, hindering efficient tissue perfusion. As a result, tissue homeostasis remains compromised, prolonging the healing process and increasing the risk of complications, such as infections and ulcer progression.

The lower signal amplitude, associated with the circuit model with equal resistances, illustrates the reduced energy contribution and additional difficulty encountered in diabetic capillary flow. As shown in Figure 12, the flow in response to a unit step input is significantly lower than others. The model with the ABI = 1.14 has a lower peripheral resistance, which results in a higher flow in the graph in Figure 12. This increase in resistance can be translated into clinical practice as a direct impact on the efficiency of gas and nutrient exchange in tissues, leading to complications such as tissue hypoxia and accumulation of metabolic products, conditions common in patients with diabetes.

Relating this to the physiological system of a capillary, in a healthy capillary, the resistance to blood flow and the energy storage capacity is balanced, allowing the efficient exchange of oxygen and nutrients, which is essential for healthy tissue repair.

In this context, ω (Equation (19)) represents the angular frequency related to the heart rate, which introduces a sinusoidal input to the circuit. This sinusoidal input simulates the pulsatile nature of blood flow driven by the heart’s rhythmic contractions. The interplay of $R_{1}$, $R_{2}$, and the capacitive reactance at different frequencies significantly influences the overall impedance and, consequently, the system’s dynamic response.

If the resistance $R_{2}$ is reduced relative to $R_{1}$, this may symbolize areas of lower resistance due to the formation of microaneurysms or selective capillary damage. The increase in the capacitor value may reflect a higher capacity of the tissue to store fluids or capillary dilation due to inflammation or edema.

These changes result in a greater output signal amplitude, suggesting a more intense energetic response that, in the physiological context, may represent the body’s attempt to compensate for inefficiency in blood flow through damaged capillaries. This compensation can lead to increased local pressure and the exacerbation of microvascular complications, common in patients with diabetes, such as diabetic retinopathy and nephropathy.

This behavior is analogous to what is observed in electrical systems, where changes in resistance and capacitance can modify the amplitude and phase of the response to the input signal. In the physiological context, this reflects how alterations in the patients’ hemodynamic conditions, such as blood pressure or vascular resistance, can affect the efficiency of blood flow and, consequently, the capillary system’s ability to transport nutrients and oxygen to tissues. The state-space model, adjusted by PSO, proved effective in capturing these dynamics, allowing the quantification of changes in system parameters associated with different ABI values. Thus, PSO can be a useful tool for analyzing and predicting the behavior of the circulatory system, providing important insights for the diagnosis and monitoring of conditions such as peripheral artery disease in diabetic patients.

In diabetic patients, this suggests that the combination of high-resistance areas, due to thickening of the capillary wall, and areas of minimal resistance, possibly due to microaneurysms, can lead to inefficient perfusion, where some areas receive too much blood and others receive too little. The high capacitor value may suggest a high capacity for charge storage, which can be compared to an increased capacity for fluid storage in the capillaries. This may indicate the presence of edema or inflammation in the tissues, which is common in patients with diabetic complications. An increase in capacitance may also reflect the body’s attempt to compensate for inadequate perfusion by storing more blood in dilated capillaries. Consequently, the output signal amplitude for ABI=1.14 is higher, indicating greater energy power. The increase in amplitude can be interpreted as an exaggerated response of the capillary system to blood flow, possibly due to inadequate resistance. This can result in pressure fluctuations that further complicate tissue perfusion and healing, increasing the risk of additional vascular damage.

The steady-state error in a control system can be analogized to the inability of capillaries to maintain adequate blood flow, causing edema and increased local blood pressure. The difficulty in adjusting system parameters is similar to compromised capillary regulation, where an inadequate response to metabolic needs can result in capillary hypertension and tissue damage. Ultimately, performance limitations in a control system reflect capillary dysfunction, which can lead to severe conditions such as diabetic retinopathy, peripheral neuropathy, and cardiovascular diseases. This analogy highlights the importance of proper performance in control systems and physiological contexts to maintain health and functionality.

The normal ABI of Patient B suggests that no detectable ischemia is present. However, the elevated arterial pressure in the lower limbs may indicate issues related to peripheral hypertension or other vascular conditions affecting circulation, even in the absence of detectable ischemia. This may require further investigation to better understand the impact of peripheral hypertension on the patient’s vascular health.

This configuration implies a distinct system dynamic, reflecting particular physiological characteristics. We can relate these values to the physiological capillary system of a diabetic patient: a high value of $R_{1}$ suggests significant resistance to flow in one part of the system. In contrast, a very low value of $R_{2}$ indicates almost no resistance in another part. This part is connected in parallel with a capacitor, which in this case showed a value higher than the others.

### 4.1. Analysis of Capillary Flow and Pressure

Previous research has investigated various microvascular parameters, such as capillary blood flow and pressure in affected areas, with the aim of better understanding how these complications impact the dynamics of blood in small vessels. However, the results of different studies have been inconsistent, with some finding no significant differences between patients with ulcers and those with other complications but without ulcers. The present study aims to contribute to this area of research by offering a detailed analysis of the microvascular factors involved, including capillary resistance, and their relationship with the development and healing of foot ulcers in diabetic patients.

In relation to variables such as blood flow, previous studies have shown no significant differences in microvascular flow or pressure parameters between patients with foot ulcers and those with other complications but without ulcers. Although diabetic patients with complications demonstrated a general reduction in capillary blood cell velocity (CBV) and laser Doppler fluxmetry (LDF), the presence of foot ulcers did not significantly affect microvascular flow or pressure measurements. For example, peak CBV was 0.14 ± 0.07 mm/s in patients with complications but no ulcers and 0.18 ± 0.14 mm/s in patients with ulcers, both significantly lower than in the control group (p<0.01). Similarly, peak LDF was 2.7 ± 2.0 V in patients without ulcers and 3.8 ± 2.8 V in those with ulcers, with no statistically significant difference observed between the two groups. These findings suggest that, within the context of this study, the presence of foot ulcers did not notably influence the dynamics of microvascular blood flow. This implies that factors such as glycemic control and neuropathy may play a more crucial role in the development and healing of ulcers than microvascular changes related to blood flow [54].

Another study investigated the skin microcirculation in the toenail area of twenty diabetic patients (sixteen with type 2 diabetes and four with type 1 diabetes) and twenty non-diabetic controls, who were matched for age and blood pressure. The average duration of diabetes in the patients was 20 ± 16 years. Among the diabetic patients, nine had foot ulcers and resting pain, while another nine had only intermittent claudication. Microcirculatory parameters such as CBV and LDF were assessed. The results showed that peak CBV was significantly reduced in diabetics compared to healthy controls (p<0.01), with 11 diabetic patients showing no post-occlusive hyperemia response in their capillaries. The percentage increase in CBV (CBV %) was considerably lower in diabetics (p<0.001) compared to healthy controls, though no significant difference was found between diabetic patients with and without ulcers. LDF analysis also revealed significantly lower LDF % values in diabetics (p<0.05) compared to controls. These results suggest that blood flow distribution is impaired in the microcirculation of diabetic patients, potentially disrupting nutrient and oxygen exchange in the feet [54].

An analysis of capillary flow and pressure was conducted in 40 insulin-dependent diabetic patients with neuropathy, 20 diabetic patients without neuropathy, and 20 healthy controls. Microcirculation was evaluated using LDF, a technique for measuring capillary blood flow and vasomotor response. Results revealed significant differences between the groups. Diabetic patients with neuropathy had a mean baseline LDF flow of 26.2 ± 2.2 perfusion units (pu), significantly higher than diabetic patients without neuropathy (16.1 ± 2.0 pu) and healthy controls (18.6 ± 2.8 pu). During the inspiratory gasp test, the percentage decrease in capillary flow was significantly lower in patients with neuropathy (27.8%) compared to those without neuropathy (47.8%) and controls (51.3%). In the post-occlusive hyperemia (PRH) test, patients with neuropathy showed the lowest percentage increase in LDF (174.7 ± 29.1%), while patients without neuropathy and controls showed significantly higher LDF increases, at 287.8 ± 40.7% and 384.3 ± 65.1%, respectively. These findings suggest alterations in vasomotor response and capillary perfusion in diabetics with neuropathy, indicative of microcirculatory dysfunction associated with the disease [55].

When comparing the values found in our study with those from previous research, several key differences and similarities emerge. Previous studies on capillary flow and pressure in diabetic patients with complications such as ulcers showed that although CBV and LDF were reduced in diabetics, no significant differences were observed between patients with and without ulcers in terms of microvascular parameters. For instance, the study by [54] observed a CBV of 0.18±0.14 mm/s in patients with ulcers, which was lower than the value observed in the control group. In contrast, our study found that capillary flow in patients (ranging from 1.95 × 10^−13^ m^3^/s to 3.90 × 10^−13^ m^3^/s) remained within expected ranges, but capillary resistance was significantly higher, reaching 1.43 × 10^16^ Pa·s/m^3^, suggesting a more pronounced impact on microcirculation than the flow values alone would indicate [54].

Furthermore, while LDF studies reported values between 2.7 ± 2.0 V and 3.8 ± 2.8 V, our simulation data revealed higher capillary pressures, ranging from 20.83 mmHg to 41.67 mmHg, compared to the expected average of 25 mmHg. Notably, Patient C had a capillary pressure of 41.67 mmHg, suggesting a disruption in hemodynamic balance, potentially due to the peripheral hypertension observed (200 mmHg systolic blood pressure). The most significant difference, however, was the consistently high capillary resistance observed in all patients, indicating a more pronounced microcirculatory dysfunction than what previous studies have shown.

These findings suggest that while capillary flow may not be drastically affected in terms of velocity or intensity in patients with ulcers, elevated capillary resistance could be a crucial factor in altering flow and contributing to the development of complications like ulcers. This highlights the importance of considering capillary resistance, along with flow and pressure, for a more comprehensive understanding of microcirculation in diabetic patients.

### 4.2. Future Directions

As evidenced by the patient profiles in this study and supported by the existing literature, the ABI values not only reflect the presence of peripheral artery disease but also serve as a predictive marker for wound healing, enabling more targeted and effective management. By combining the ABI with engineering techniques, we propose a cost-effective solution for these patients, offering a practical and accessible approach. However, it is crucial to note that this approach requires further investigation and validation in multicenter studies to refine its applicability and confirm its effectiveness across diverse patient populations and clinical settings.

Peripheral neuropathy, a common condition in diabetic patients, can lead to increased plantar pressure and delayed healing due to the loss of protective sensation. Additionally, elevated blood glucose levels negatively affect angiogenesis and the inflammatory response, further hindering tissue repair processes. We also plan to conduct future experimental validations in larger cohorts to assess whether the trends identified in this study hold true in a broader patient population. While our research focused on the relationship between ABI and ulcer size, we acknowledge that other factors, such as peripheral neuropathy, glycemic control, and systemic inflammation, may also play significant roles in the healing process.

Another measurement of Laser Doppler Fluxometry (LDF) in the pulp area of the big toe showed that the DM group (diabetes without neuropathy) had an average perfusion of 161.08 ± 100.51 arbitrary units. This is significantly higher than the DU group (diabetes with neuropathy and ulcer), which had 139.46 ± 79.23 units, and the healthy control group, which had 93.53 ± 63.26 units (*p* = 0.009). These data indicate that the presence of neuropathy and diabetic ulcers results in a decrease in capillary blood perfusion, highlighting a microcirculatory dysfunction in patients with diabetic neuropathy complications. Moreover, epidermal thickness was significantly different between the groups, with the DU group showing a reduced thickness, while the DM group showed an increase when compared to controls [56].

## 5. Conclusions

This study provided an initial analysis of the relationship between ABI and ulcer healing in diabetic patients, using a mathematical model. The findings demonstrate that variations in hemodynamic parameters can significantly influence tissue perfusion, reinforcing the importance of integrated approaches to assess microcirculation. Although the small sample size limits the generalization of the results, the proposed model represents a first step toward a quantitative assessment of microcirculation in diabetic ulcers. Future studies should validate the results with larger experimental datasets, incorporating factors such as neuropathy and systemic inflammation for a more comprehensive analysis. Future studies should integrate these variables into the model, allowing for a broader analysis of the interactions between microcirculation, glycemic metabolism, and inflammatory factors in the healing of diabetic ulcers.

## Figures and Tables

**Table 1:** Description of the components and their influence on system dynamics.

Component	Description
*R_1_*	Resistance influences the energy dissipation in the system, affecting the damping rate of the initial dynamic responses.
*R_2_*	Additional resistance influences the energy dissipation in the system, affecting the damping rate of subsequent dynamic responses.
*C*	The capacitor influences the system’s ability to store electrical energy. It affects the state matrix, particularly in the terms that represent the system’s ability to respond to state changes, altering the natural frequencies and modes of oscillation.
*L*	The inductor influences the system’s inductance, affecting the transient response. It impacts the state and output matrices, modifying the transfer function and influencing the system’s stability through the modes of dynamic response.

**Table 2:** Physical and physiological quantities (ABI = 1) of arterial (PTA) and capillary vessels used in the study.

Parameter	Artery (PTA)	Capillary	References
Diameter (mm)	3	0.007	[40,41,42]
Radius (mm)	1.25	0.003	[40,41,42]
Length (m)	0.05	0.0007	[43,44]
Thickness (mm)	0.5	0.0005	[40,42]
Pressure (mmHg)	120	25	[42,45]
Density (kg/m^3^)	1060	1060	[39]
Viscosity (Pa·s)	0.0045	0.0012	[39,46,47]
Flow (m^3^/s)	$5.30\times 10^{-4}$	$2.34\times 10^{-13}$	Calculated [39]
Resistance (Pa·s/m^3^)	$3.02\times 10^{7}$	$1.43\times 10^{16}$	Calculated [39]

Legend: PTA: posterior tibial artery; mm: millimeters; m: meters; mmHg: millimeters of mercury; kg/m^3^: kilograms per cubic meter; Pa·s: Pascal seconds; m^3^/s: cubic meters per second; Pa·s/m^3^: Pascal seconds per cubic meter.

**Table 3:** Flow in the blood capillary calculated from the ABI based on Poiseuille’s Law, which describes the flow of viscous fluids under a laminar regime.

ABI Index	**Q (m^3^/s)**
0.6	$1.94856\times 10^{-13}$
1.14	$3.11769\times 10^{-13}$
1.33	$3.89712\times 10^{-13}$

**Table 4:** Variables analyzed for integration with the computer system.

Patient Code	Pressure (mmHg)		ABI Index	Wound Size (${\mathrm{cm}}^{2}$)
Patient A	160	100	0.6	3.978
Patient B	140	160	1.14	0.357
Patient C	150	200	1.33	6.038

Legend: Patient Code: identifier for each patient; Pressure (mmHg): measurement of systolic blood pressure in the upper and lower limbs, respectively; ABI Index: ankle–brachial index; Wound Size (cm^2^): size of the wound in square centimeters.

**Table 5:** Parameter values from PSO.

$R_{1}$	$R_{2}$	*L*	*C*
89.784	426.55	27.506	0.00040675

## Data Availability

The raw data and scripts that support the conclusions of this article are available at the Supplementary Materials.

## Supplementary Materials

The following supporting information can be downloaded at: https://www.mdpi.com/article/10.3390/bioengineering12020206/s1. Figure S1: Flow vs. Pressure for Patient with ABI = 0.6. Figure S2: Varying Parameters for Patient with ABI = 0.6. Figure S3: Flow vs. Pressure for Patient with ABI = 1.14. Figure S4: Varying Parameters for Patient with ABI = 1.14. Figure S5: Flow vs. Pressure for Patient with ABI = 1.33. Figure S6: Varying Parameters for Patient with ABI = 1.33. Table S1: Automatic Calculation Table for Blood Capillary Parameters. File S1: MATLAB code used in the article.
