# Supplementary figures and images for: Development of Mathematical Model for Understanding Microcirculation in Diabetic Foot Ulcers Based on Ankle–Brachial Index
Source: Bioengineering (Basel). 2025 Feb 19;12(2):206. doi: 10.3390/bioengineering12020206 (PMC11851477; doi:10.3390/bioengineering12020206)

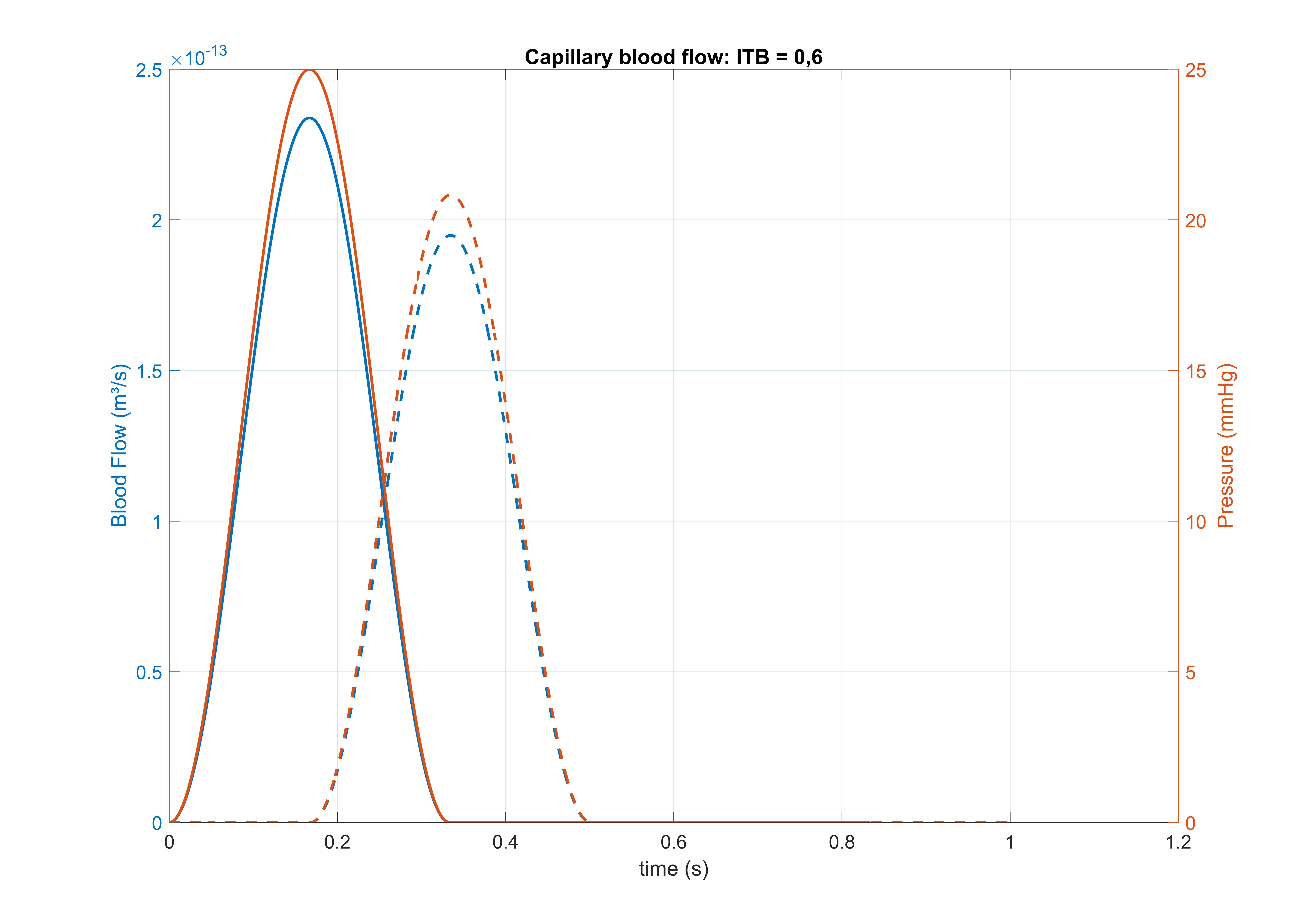

Supplement: Supplementary file 1 [file bioengineering-12-00206-s001.zip › 1. Patient ABI = 0.6/FlowVsPressure06.jpg]

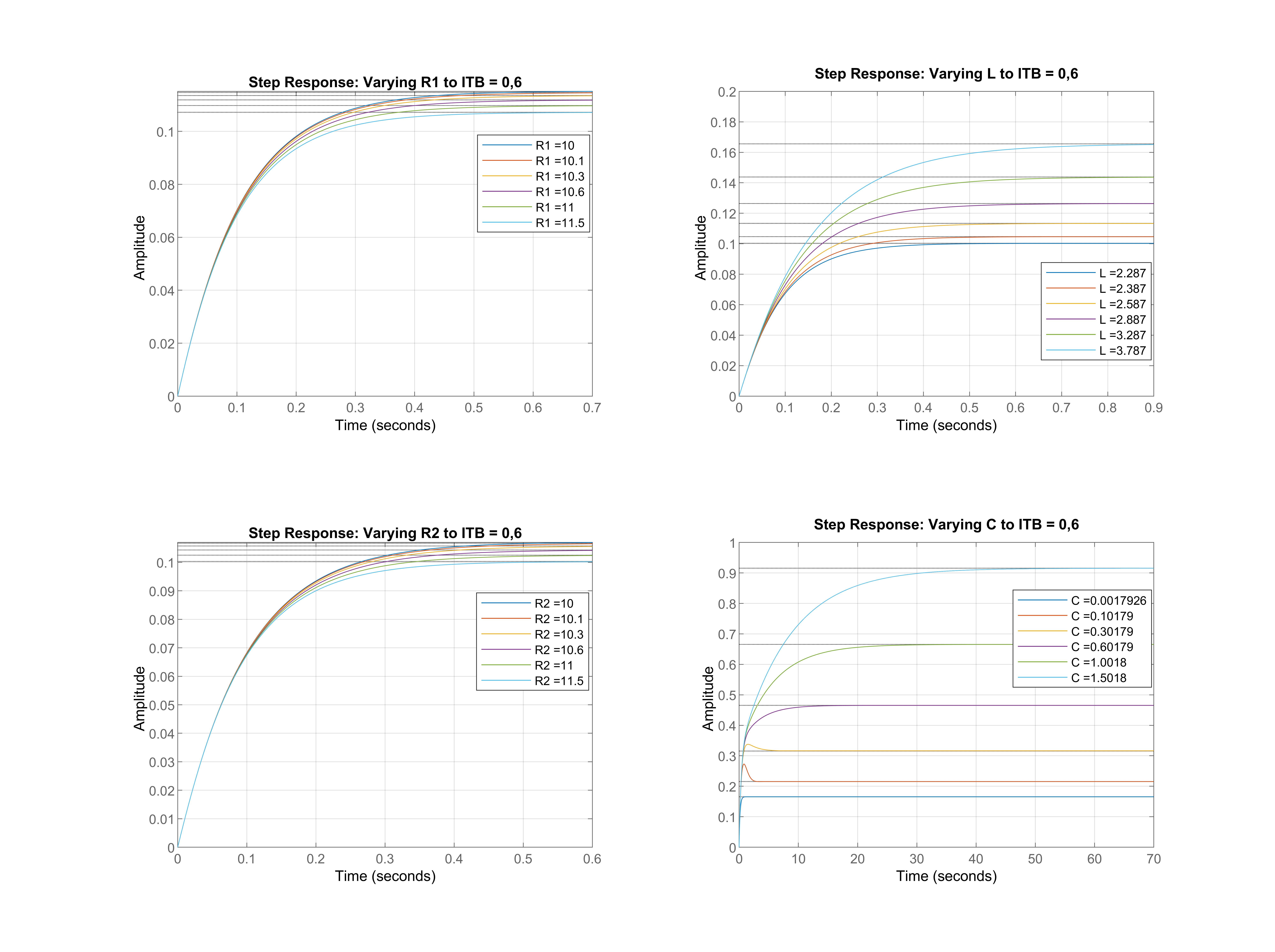

Supplement: Supplementary file 1 [file bioengineering-12-00206-s001.zip › 1. Patient ABI = 0.6/VaryingParameters06.jpg]

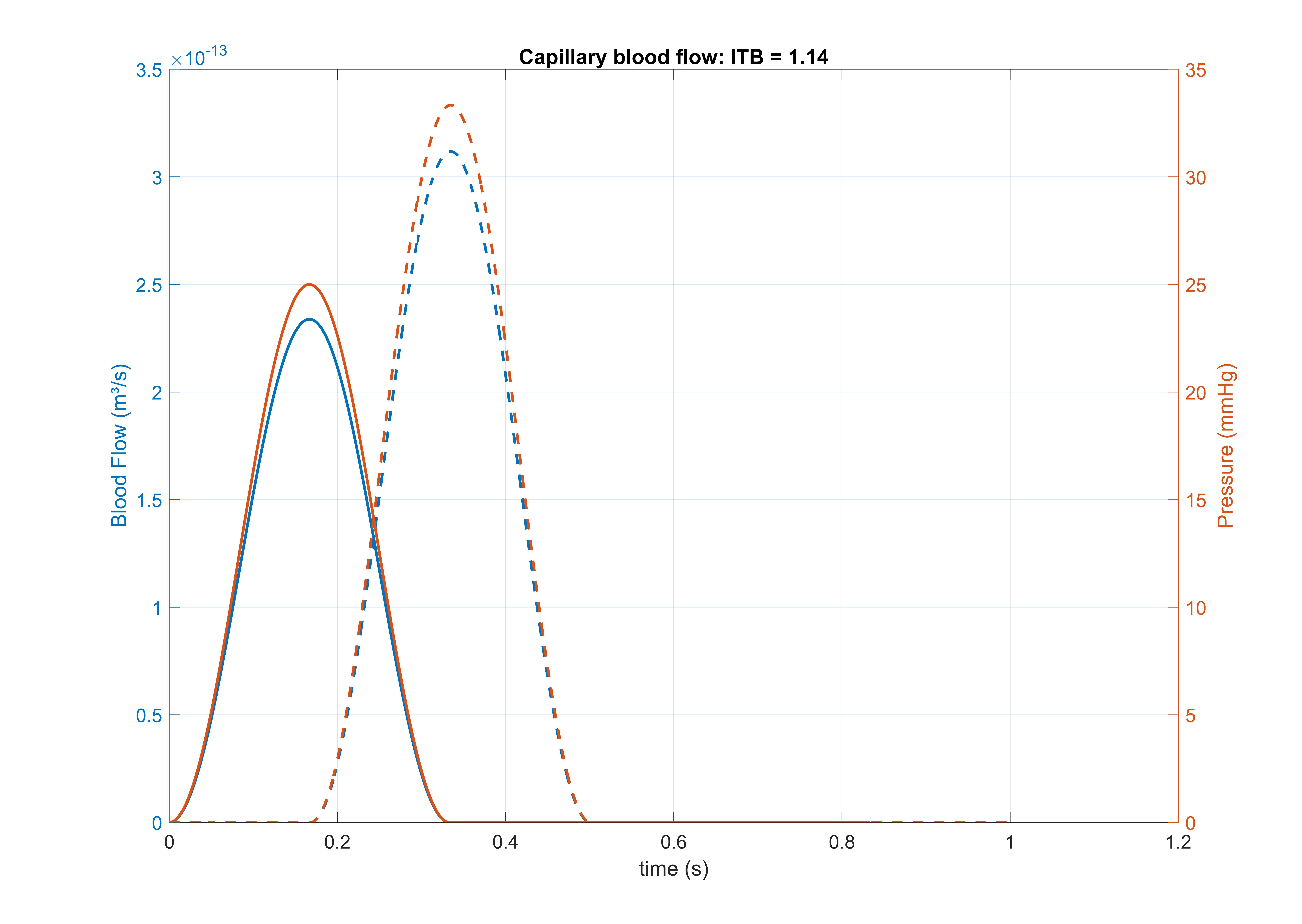

Supplement: Supplementary file 1 [file bioengineering-12-00206-s001.zip › 2. Patient ABI = 1.14/FlowVsPressure114.jpg]

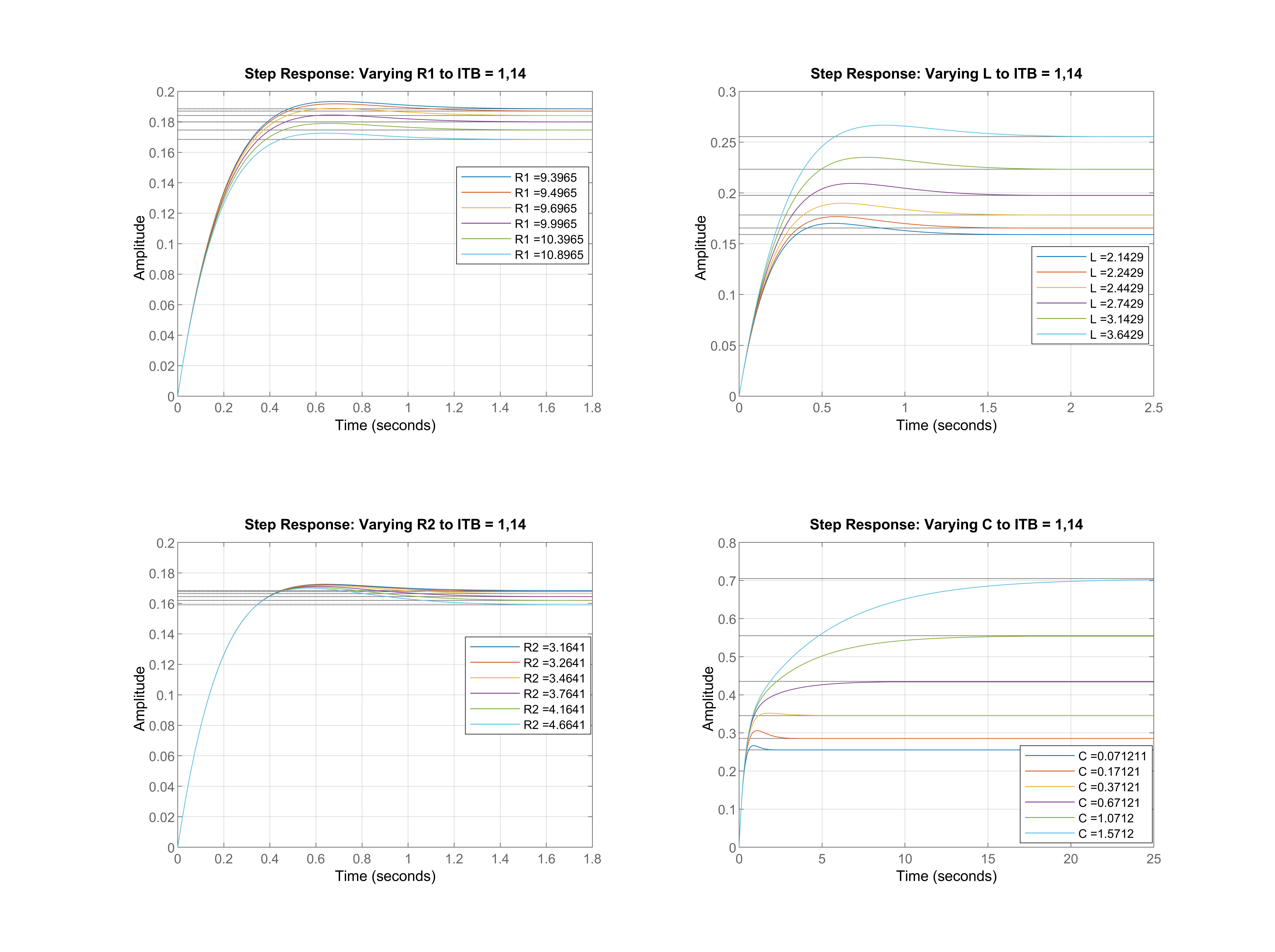

Supplement: Supplementary file 1 [file bioengineering-12-00206-s001.zip › 2. Patient ABI = 1.14/VaryingParameters114.jpg]

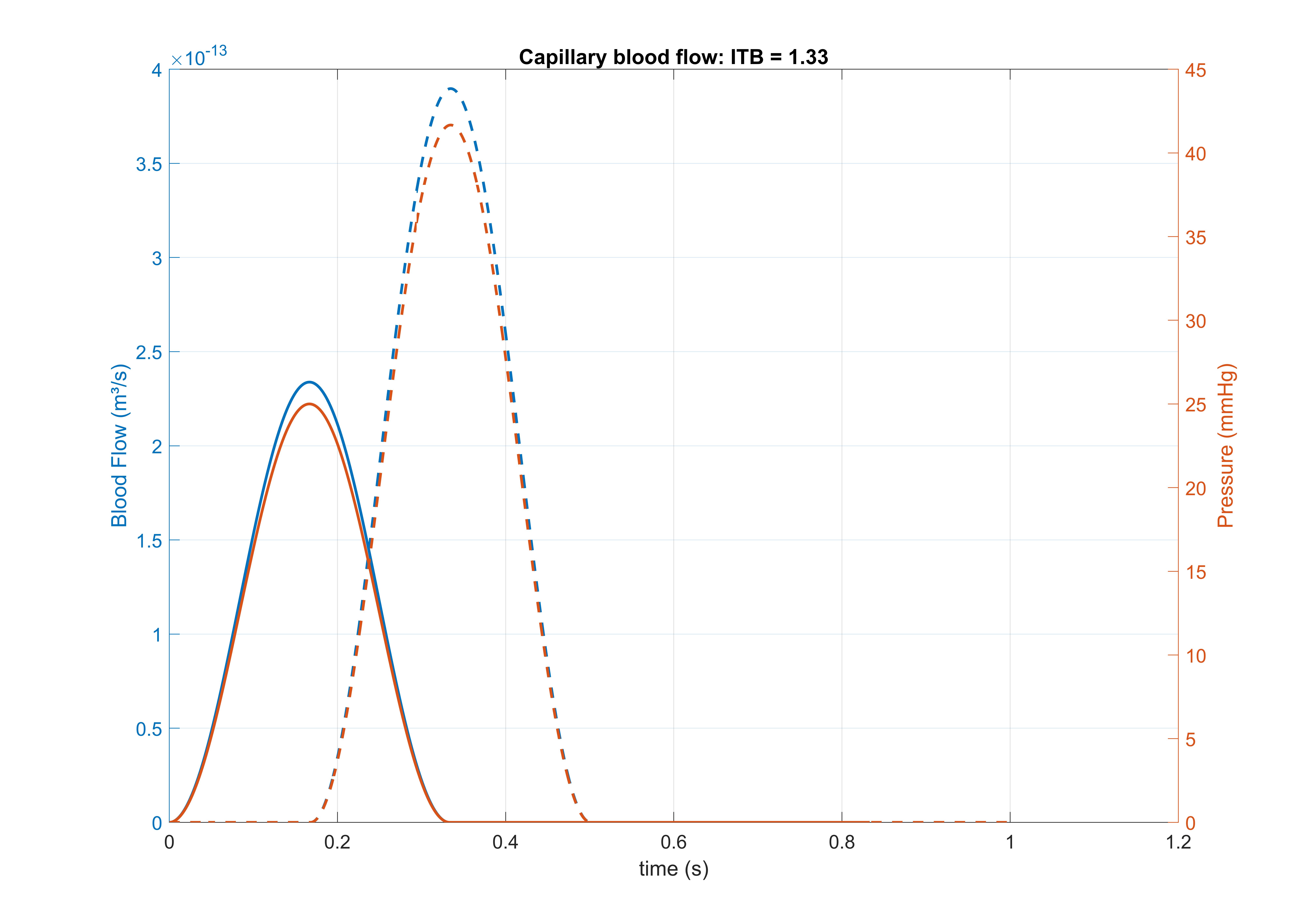

Supplement: Supplementary file 1 [file bioengineering-12-00206-s001.zip › 3. Patient ABI = 1.33/FlowVsPressure133.jpg]

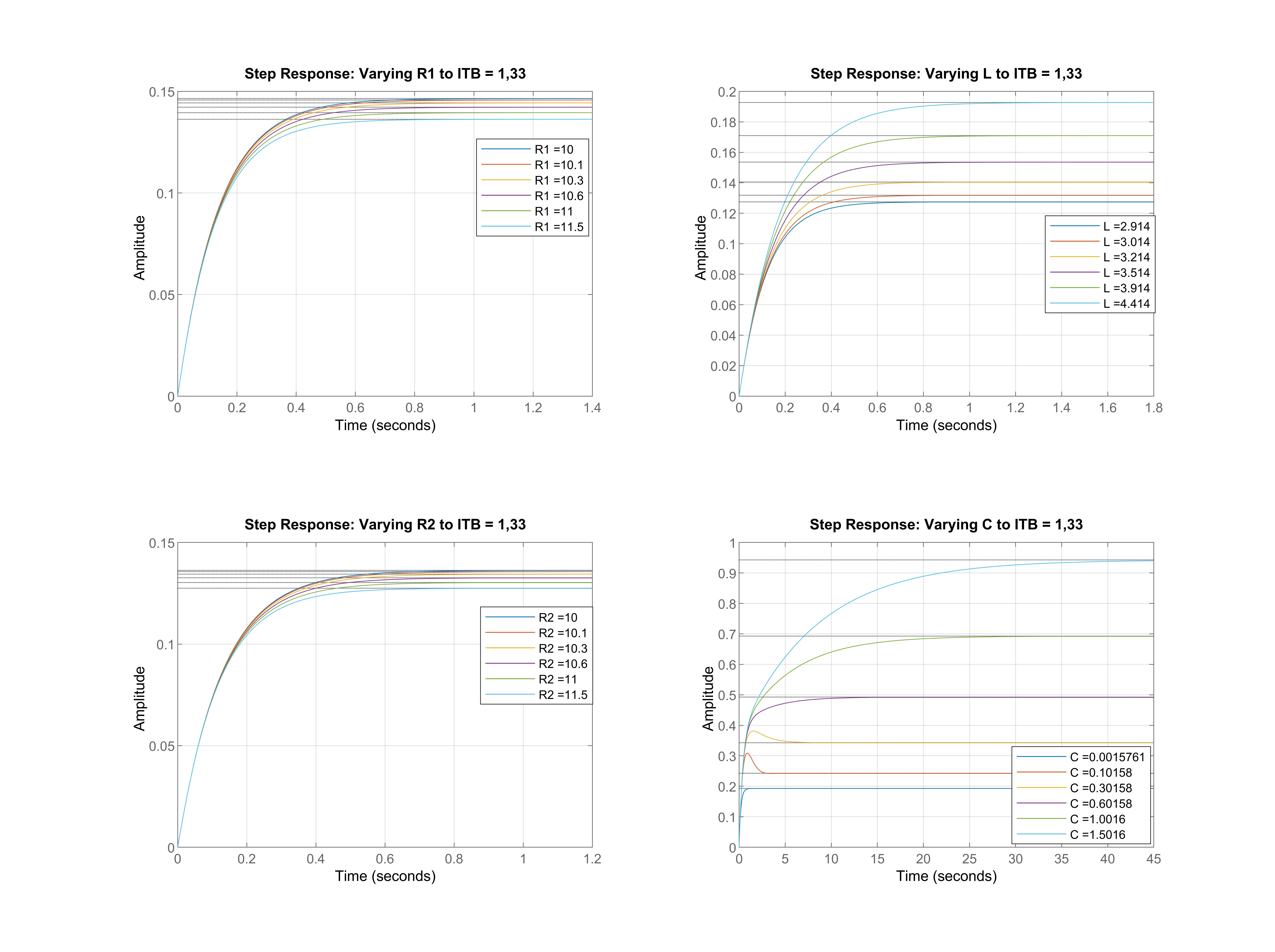

Supplement: Supplementary file 1 [file bioengineering-12-00206-s001.zip › 3. Patient ABI = 1.33/VaryingParameters133.jpg]
